# Lactylation‐Driven YTHDC1 Alleviates MASLD by Suppressing PTPN22‐Mediated Dephosphorylation of NLRP3

**DOI:** 10.1002/advs.202510192

**Published:** 2025-11-26

**Authors:** Feng Zhang, Linghua Zeng, Kunkun Zou, Keying Lin, Fangting Yao, Jinglun Song, Shumeng Zhang, Hanshu Feng, Zhichao Yang, Chunlei Wang, Hongtao Diao, Xue Kong, Tengfei Pan, Joonki Kim, Tianqi Duo, Linqiang Li, Yu Bian

**Affiliations:** ^1^ General Surgery Department The Second Affiliated Hospital of Harbin Medical University Harbin Heilongjiang Province 150086 China; ^2^ Department of Pharmacology (State Key Laboratory of Frigid Zone Cardiovascular Diseases, the State‐Province Key Laboratories of Biomedicine‐Pharmaceutics of China, Key Laboratory of Cardiovascular Research, Ministry of Education), College of Pharmacy Harbin Medical University Harbin 150081 China; ^3^ Department of Minimal Invasive Hepatic Surgery The First Affiliated Hospital of Harbin Medical University Harbin Heilongjiang 150001 China; ^4^ The Academician Cooperative Laboratory of Basic and Translational Research on Chronic Diseases The First Affiliated Hospital Jinan University Guangzhou 510632 China; ^5^ Natural Product Applied Science, KIST School University of Science & Technology (UST) Gangneung 25451 Republic of Korea

**Keywords:** lactylation, MASLD, NLRP3, PTPN22, YTHDC1

## Abstract

Metabolic dysfunction‐associated steatotic liver disease (MASLD) is among the most prevalent chronic liver diseases worldwide. The expression of YTH domain‐containing protein 1 (YTHDC1) is significantly reduced in patients and mouse models with MASLD. Hepatocyte‐specific knockout of YTHDC1 exacerbates HFD‐induced hepatic lipid accumulation. Lactate accumulation and enhanced arginyl‐tRNA synthetase 1 (AARS1)‐mediated K565‐specific lactylation are shown to drive the ubiquitination‐mediated degradation of YTHDC1. This work proposes that under MASLD conditions, diminished YTHDC1‐LDHA binding elevates free LDHA levels, which enhances YTHDC1 lactylation and suppresses its expression. This creates a positive feedback loop that exacerbates the progression of MASLD. Mechanistically, protein tyrosine phosphatase nonreceptor type 22 (PTPN22), identified as a downstream target of YTHDC1, exacerbates hepatic inflammation and lipid accumulation by dephosphorylating and activating NLRP3 at tyrosine 861, which in turn promotes the release of IL‐1β and IL‐18. Furthermore, mebendazole, a small‐molecule drug targeting YTHDC1, significantly alleviates MASLD. In conclusion, YTHDC1 mitigates MASLD by inhibiting the PTPN22‐mediated dephosphorylation and activation of NLRP3, offering new insights into therapeutic strategies for MASLD.

## Graphical abstract

In MASLD, YTHDC1 undergoes increased lactylation and ubiquitination, reducing its expression. AARS1 mediates lactylation at lysine 565, while disrupted binding to LDHA further promotes lactylation, suppressing YTHDC1. This downregulation enhances PTPN22 mRNA stability, leading to NLRP3 dephosphorylation and activation, which exacerbates inflammation and lipid accumulation. The study suggests mebendazole as a potential treatment targeting YTHDC1 in MASLD.

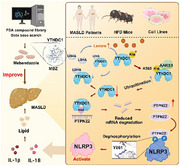

## 1 Introduction

Metabolic dysfunction‐associated steatotic liver disease (MASLD), formerly known as nonalcoholic fatty liver disease (NAFLD), is the most prevalent chronic liver disease worldwide, affecting ≈20%–30% of the global population.^[^ ^1^ ^]^ The new “positive criteria” for MASLD, which were proposed by an international panel of experts from 22 countries, are based on the presence of liver steatosis and one of the following: overweight/obesity, type 2 diabetes, and metabolic dysregulation.^[^ ^2^ ^]^ No specific therapeutic drugs are currently available for MASLD, and the optimal balance between cost and treatment duration remains uncertain.^[^ ^3^ ^]^

N^6^‐methyladenosine (m^6^A) is a major posttranslational modification that regulates messenger RNA (mRNA) stability in various biological processes.^[^ ^4^ ^]^ It involves multiple methyltransferase complexes such as writers, erasers, and readers. m6A reader proteins play essential roles in both the recognition and binding of m6A‐modified RNAs, thereby influencing mRNA stability and splicing, export, and translation efficiency. On the basis of their localization, m6A readers include nuclear readers such as YTH domain‐containing protein 1 (YTHDC1), heterogeneous nuclear ribonucleoprotein A2/B1 (HNRNPA2B1), heterogeneous nuclear ribonucleoprotein C (HNRNPC11), and heterogeneous nuclear ribonucleoprotein G (HNRNPG) and cytoplasmic readers such as YTH domain‐containing family proteins 1, 2, and 3 (YTHDF1/2/3), YTH domain‐containing protein 2 (YTHDC2), and insulin‐like growth factor 2 mRNA‐binding proteins 1, 2, and 3 (IGF2BP1/2/3).^[^ ^5^ ^]^ Uniquely, YTHDC1 is the only reader in the YTH family and is localized in the nucleus.^[^ ^6^ ^]^ Previous studies have shown that YTHDC1 interacts with splicing factors and regulates mRNA splicing,^[^ ^7^ ^]^ transcription,^[^ ^8^ ^]^ and nuclear export.^[^ ^9^ ^]^ In addition, YTHDC1 is closely associated with chromatin, noncoding RNAs, and regulatory RNAs and plays a pivotal role in transcriptional regulation and the regulation of gene expression.^[^ ^10^ ^]^ More recently, various functions of YTHDC1 have been reported in tumors, including breast, lung,^[^ ^11^ ^]^ bladder,^[^ ^12^ ^]^ and ovarian cancer.^[^ ^13^ ^]^ The stability of downstream mRNA mediated by YTHDC1 has also been demonstrated in diabetes, lung injury, and cancer.^[^ ^14^, ^15^, ^16^ ^]^ However, no studies have reported its role in promoting mRNA degradation.

Traditionally, lactate has been viewed as a waste product of glucose metabolism that is excreted and discarded by cells.^[^ ^17^ ^]^ However, lactate‐derived lactylation has been recently identified as a post‐translational modification. Studies have shown that lactate is absorbed by tumor cells and transported into the mitochondria for oxidation to generate energy while also inducing lactylation of histone lysine residues, thereby stimulating gene transcription.^[^ ^18^ ^]^ The abundance and specificity of non‐histone proteins in cells are higher than those of histones. However, previous studies have not clarified whether non‐histone proteins undergo notable lactylation, nor have they elucidated the functioning of these lactylated non‐histone proteins as well as their ability to regulate tumor progression.^[^ ^19^ ^]^

Protein tyrosine phosphatase nonreceptor type 22 (PTPN22) belongs to the protein tyrosine phosphatase family and plays a critical role in modulating T cell signaling.^[^ ^20^ ^]^ The *PTPN22* gene is located on chromosome l p13.2 and encodes cytoplasmic lymphoid phosphatase (LYP), which is essential for maintaining the balance of immune system function. In recent years, the susceptibility of PTPN22 to various inflammatory diseases has been well‐documented. Studies have indicated that PTPN22 expression is restricted to hematopoietic cells.^[^ ^21^ ^]^ Additionally, PTPN22 expression has been shown to be significantly associated with the risk of idiosyncratic drug‐induced liver injury (DILI).^[^ ^22^ ^]^ However, the participation of PTPN22 in regulating the MASLD pathway remains unclear and warrants further investigation.

In the present study, we demonstrated that the m^6^A reader YTHDC1 targets and interacts with the m^6^A‐modified Ptpn22 mRNA, facilitating its degradation. Hepatocyte‐specific overexpression of Ythdc1 mitigated the progression of MASLD by inhibiting the tyrosine phosphorylation and activation of NLR family pyrin domain containing 3 (NLRP3). These findings indicate that YTHDC1 is a promising therapeutic target for the treatment of MASLD.

## 2 Results

### 2.1 Downregulation of YTHDC1 in MASLD

**Figure 1:**
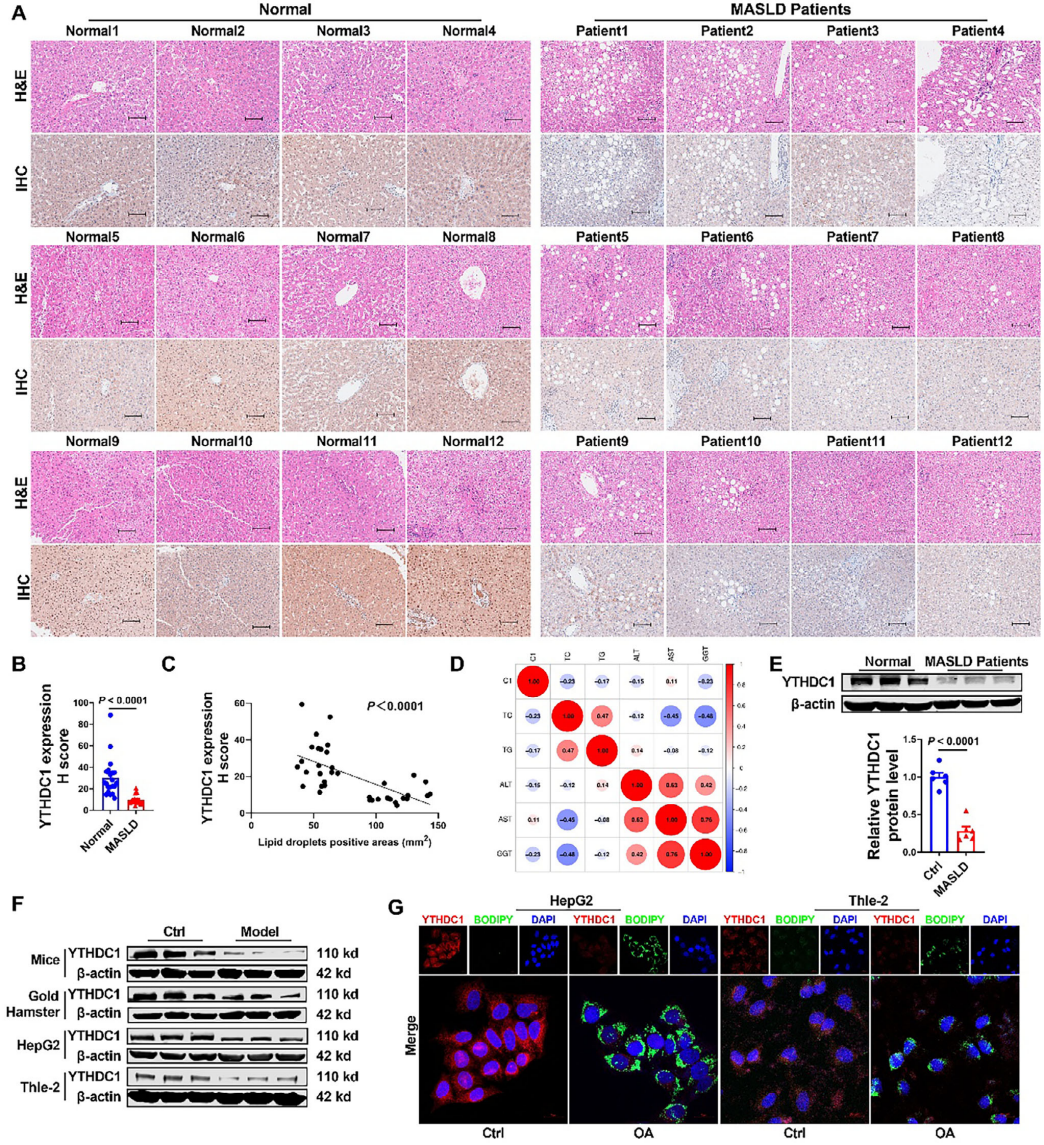
YTHDC1 is downregulated in MASLD. A) Representative immunohistochemical and H&E staining of liver tissue of patients with MASLD and normal donor liver tissue. Scale bars=100 µm. *n* = 16–21. B) Representative statistics of immunohistochemical staining of liver tissue and normal donor liver tissue in MASLD patients. *n* = 16–21. C) Analysis of the correlations between YTHDC1 level and lipid droplet‐positive areas. *n* = 16–21. D) Heatmap for correlation analysis of YTHDC1 and TC, TG, ALT, AST, GGT levels in plasma of healthy controls and MASLD patients. *n* = 16–21. E) Western blot analysis of YTHDC1 protein levels in MASLD patients and normal individuals. *n* = 6. F) Western blot analysis of YTHDC1 protein levels in HFD‐induced mice and golden hamster groups and at 24 h after OA induction in HepG2 and Thle‐2 cells. *n* = 6. G) Immunofluorescence and BODIPY representative images of YTHDC1 expression in oleic acid‐induced HepG2 and Thle‐2. Scale bars=100 µm. *n* = 4. Data are presented as mean ± SEM.

We analyzed YTHDC1 expression levels in pathological liver samples from patients with MASLD and healthy donors. Immunohistochemical staining revealed a significant reduction in YTHDC1 expression in the liver tissues of patients with MASLD in comparison with that in normal donors (**Figure** **1**A,B; Figure S1A, Supporting Information). Further investigation revealed that YTHDC1 expression was negatively correlated with lipid droplet accumulation as well as cholesterol and triglyceride (TG) levels (Figure 1C,D). Western blot analysis further confirmed that YTHDC1 protein expression was significantly lower in the liver tissues of patients with MASLD than in normal tissues (Figure 1E).

Similarly, in mice and golden hamsters that received a high‐fat diet (HFD), YTHDC1 protein levels were significantly lower than those in the control group (Figure 1F; Figure S1C,D, Supporting Information). In vitro, after 24 h of exposure to oleic acid (OA), YTHDC1 protein levels were markedly decreased in HepG2, Thle‐2, and L02 cells (Figure 1F; Figure S1B,E,F, Supporting Information). Immunofluorescence and BODIPY staining revealed a significant reduction in YTHDC1 expression in the liver tissues of MASLD mice and cells models (Figure 1G; Figure S1G–P, Supporting Information). Simultaneously, we discovered that YTHDC1 is highly expressed in hepatocytes (Figure S1Q,R, Supporting Information). These findings indicated that YTHDC1 expression was downregulated during the progression of MASLD.

### 2.2 Modulating the Expression of YTHDC1 in Mice Significantly Influenced the Progression of MASLD

**Figure 2:**
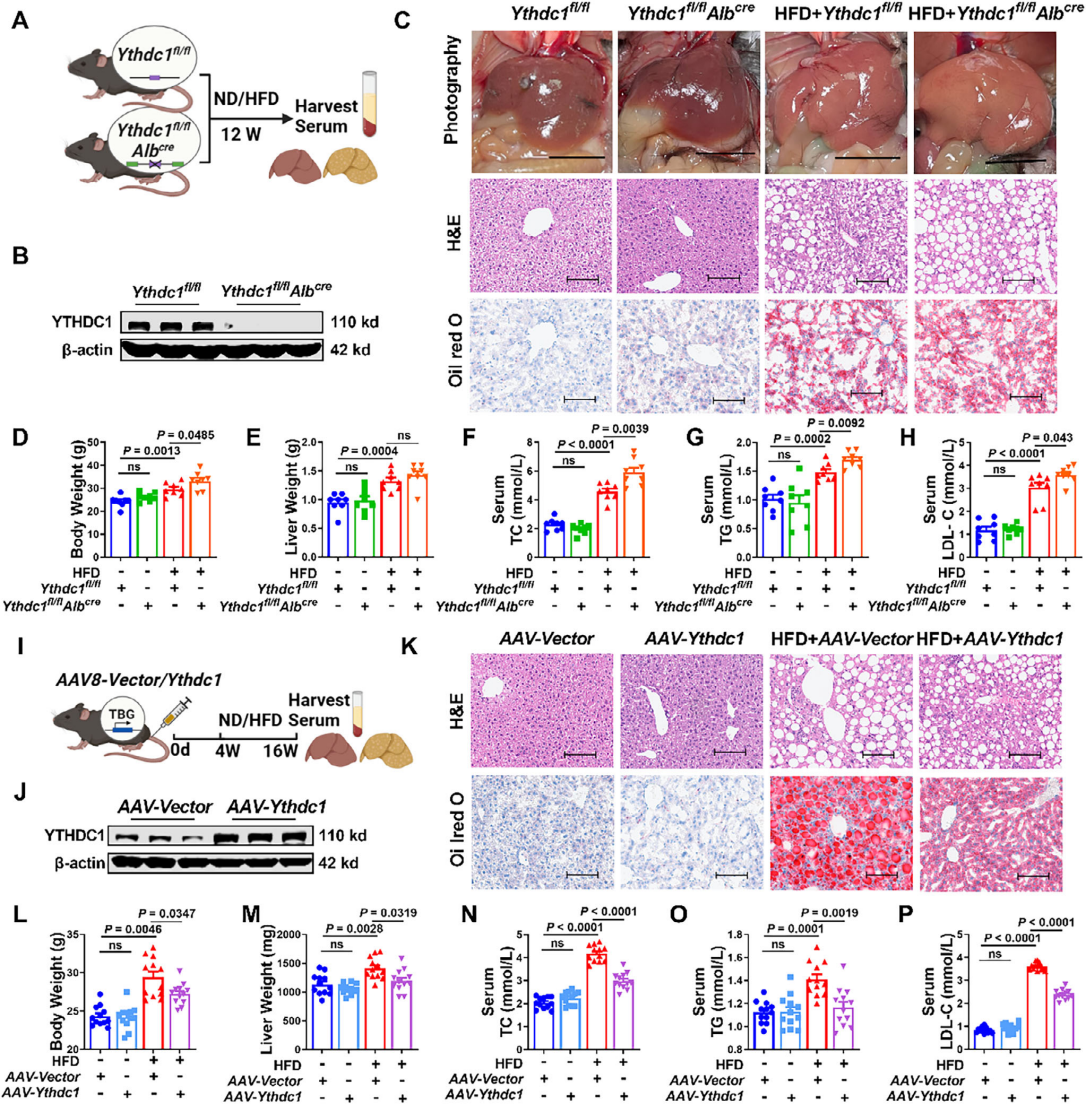
Interventions targeting the expression of YTHDC1 regulate the progression of MASLD. A) Schematic diagram of liver‐specific knockout of *Ythdc1* in mice. B) Western blot analysis of YTHDC1 levels in the liver of *Ythdc1^fl/fl^* and *Ythdc1 ^fl/fl^Alb^cre^* mice. *n* = 3. C) Representative images of HE staining and Oil Red O staining of liver images and liver sections of *Ythdc1^fl/f^* and *Ythdc1^fl/fl^Alb^cre^* mice after HFD feeding. Scale bars=100 µm. *n* = 4. D,E) The liver and body weights of *Ythdc1^fl/fl^* and *Ythdc1^fl/fl^Alb^cre^* mice after HFD induction. *n* = 8. F–H) After 12 weeks of HFD induction, the levels of TC, TG and LDL‐C level in serum of *Ythdc1^fl/fl^* and *Ythdc1^fl/fl^Alb^cre^* mice. *n* = 8. I) Process diagram for mouse tail vein injection of adeno‐associated virus expressing YTHDC1 *AAV‐Ythdc1*. J) Western blot analysis of YTHDC1 protein level in the liver of *AAV‐NC* and *AAV‐Ythdc1* mice. *n* = 3. K) For histopathological studies, liver tissue sections were stained with H&E and Oil Red O. Scale bars=100 µm. *n* = 4. L,M) The liver weight and body weight in indicated groups (*AAV‐NC*, *AAV‐Ythdc1*, HFD+*AAV‐NC*, HFD+*AAV‐Ythdc1*). *n* = 12. N–P) Statistical data of TC, TG and LDL‐C contents in serum samples. *n* = 12. Data are presented as mean ± SEM.

To investigate the role of YTHDC1 in MASLD progression, we generated liver‐specific conditional knockout (CKO) mice for YTHDC1 (**Figure** **2**A). In comparison with *Ythdc1^fl/fl^* mice, *Ythdc1^fl/fl^Alb^cre^* mice showed significantly lower YTHDC1 mRNA and protein expression levels (Figure 2B; Figure S2A,B, Supporting Information). Hepatic steatosis is the earliest pathological feature of MASLD,^[^ ^23^ ^]^ and HFD feeding results in a pale mouse liver appearance, increased liver volume, and a softer texture. Hematoxylin and eosin (H&E) staining revealed notable pathological changes, including prominent lipid droplets within the hepatocytes, ballooning degeneration, cell swelling, enlarged cell morphology, nuclei displaced toward the periphery, irregular cell arrangement, and inflammatory cell infiltration. Oil Red O staining showed lipid droplets of varying sizes distributed within hepatocytes, with these pathological changes being more pronounced in *Ythdc1^fl/fl^Alb^cre^* mice (Figure 2C; Figure S2C, Supporting Information). After HFD treatment, both liver and body weights increased significantly in *Ythdc1^fl/fl^* mice, with even greater increases observed in *Ythdc1^fl/fl^Alb^cre^* mice (Figure 2D,E). Total cholesterol (TC) and TG levels reflect the degree of lipid metabolism disorder in MASLD patients. Elevated TG levels are a hallmark of MASLD because TG accumulation in the liver is the primary driver of fatty liver formation. Increased TC levels, particularly low‐density lipoprotein cholesterol (LDL‐C) levels, are often associated with MASLD progression and reflect systemic abnormalities in lipid metabolism.^[^ ^24^ ^]^ Consistently, after 12 weeks of HFD feeding, both serum and tissue levels of TC and TG were significantly higher in *Ythdc1^fl/fl^Alb^cre^* mice than in *Ythdc1^fl/fl^* mice (Figure 2F,G; Figure S2D,E, Supporting Information). The levels of alanine aminotransferase (ALT) and aspartate aminotransferase (AST), markers of liver damage, were also significantly elevated in *Ythdc1^fl/fl^Alb^cre^* mice after 12 weeks of HFD exposure in comparison with those in *Ythdc1^fl/fl^* mice (Figure S2F–I, Supporting Information). Moreover, serum LDL‐C levels increased following HFD stimulation, with the liver‐specific knockout of YTHDC1 leading to higher LDL‐C levels (Figure 2H). Notably, in *Ythdc1^fl/fl^Alb^cre^* mice, HFD feeding resulted in significantly elevated levels of TC, TG, ALT, and AST in the liver tissues in comparison with those in *Ythdc1^fl/fl^* mice. These findings indicate that hepatocyte‐specific deficiency of YTHDC1 exacerbates HFD‐induced lipid accumulation, highlighting its critical role in lipid metabolism and MASLD progression.

To evaluate whether increased YTHDC1 expression could regulate the progression of MASLD, we overexpressed YTHDC1 in the liver of mice by tail vein injection of an adeno‐associated virus (AAV) expressing YTHDC1 (*AAV‐Ythdc1*) (Figure 2I). 4 weeks post‐infection, we assessed the efficiency of AAV infection and observed that both the protein and mRNA levels of YTHDC1 were significantly elevated in the liver tissues of *AAV‐Ythdc1* mice in comparison with those in *AAV‐Vector* mice (Figure 2J; Figure S2J,K, Supporting Information). Gross observations revealed that YTHDC1 overexpression alleviated the pale appearance and liver enlargement typically induced by HFD feeding. H&E staining showed that YTHDC1 overexpression reduced lipid droplet deposition, ballooning degeneration, cell swelling, and inflammatory infiltration in the liver in comparison with the corresponding findings in HFD‐fed *AAV‐Vector* mice. Similarly, Oil Red O staining revealed a significant reduction in lipid droplet accumulation in the liver tissues of *AAV‐Ythdc1* mice (Figure 2K; Figure S2L, Supporting Information).

Overexpression of YTHDC1 also mitigated the HFD‐induced increase in liver and body weights (Figure 2L,M). Furthermore, after 12 weeks of HFD feeding, serum and tissue levels of TC, TG, ALT, AST, and LDL‐C were significantly lower in *AAV‐Ythdc1* mice than in *AAV‐Vector* mice (Figure 2N–P; Figure S2M–P, Supporting Information). These findings indicate that under HFD‐feeding conditions, liver‐specific overexpression of YTHDC1 alleviates lipid accumulation and mitigates the pathological progression of MASLD.

### 2.3 YTHDC1 Ameliorates Lipid Accumulation In Vitro

We also conducted functional validation of YTHDC1 in cell lines. First, we constructed a plasmid vector carrying oe‐YTHDC1 to overexpress YTHDC1. In comparison to transfection with the empty vector, transfection with the vector carrying oe‐YTHDC1 induced stable YTHDC1 overexpression in HepG2 and Thle‐2 cells, as evidenced by significant increments in both protein and mRNA levels (Figure S3A,B,M,N, Supporting Information). To investigate the functional role of YTHDC1, HepG2 and Thle‐2 cells were treated with OA. In the vector‐transfected group, OA treatment significantly elevated the TC and TG levels. However, overexpression of YTHDC1 resulted in a marked reduction in TC and TG levels in comparison with those in the vector group (Figure S3D,E,P,Q, Supporting Information). Oil Red O staining further demonstrated that following OA stimulation, YTHDC1 upregulation alleviated lipid deposition in HepG2 and Thle‐2 cells in comparison with that in the empty vector group (Figure S3C,F,O,R, Supporting Information). These results suggested that YTHDC1 overexpression mitigated lipid accumulation and supported its functional role in regulating lipid metabolism.

Next, we constructed YTHDC1‐specific small interfering RNAs (siRNAs) and named them si‐YTHDC1. Western blotting and polymerase chain reaction (PCR) confirmed that transfection with si‐YTHDC1 significantly reduced YTHDC1 protein and mRNA levels in HepG2 and Thle‐2 cells (Figure S3G, H, S, T Supporting Information). After exposure to OA, TC and TG levels were significantly higher in the si‐YTHDC1 group than in the vector group in both HepG2 and Thle‐2 cells (Figure S3J, K, V, W Supporting Information). Oil Red O staining further revealed that si‐YTHDC1 exacerbated OA‐induced lipid deposition in HepG2 and Thle‐2 cells (Figure S3I, L, U, X, Supporting Information). Overall, these experiments demonstrated that upregulation of YTHDC1 significantly inhibited lipid accumulation, whereas its knockdown promoted lipid deposition, highlighting the critical role of YTHDC1 in lipid metabolism.

**Figure 3:**
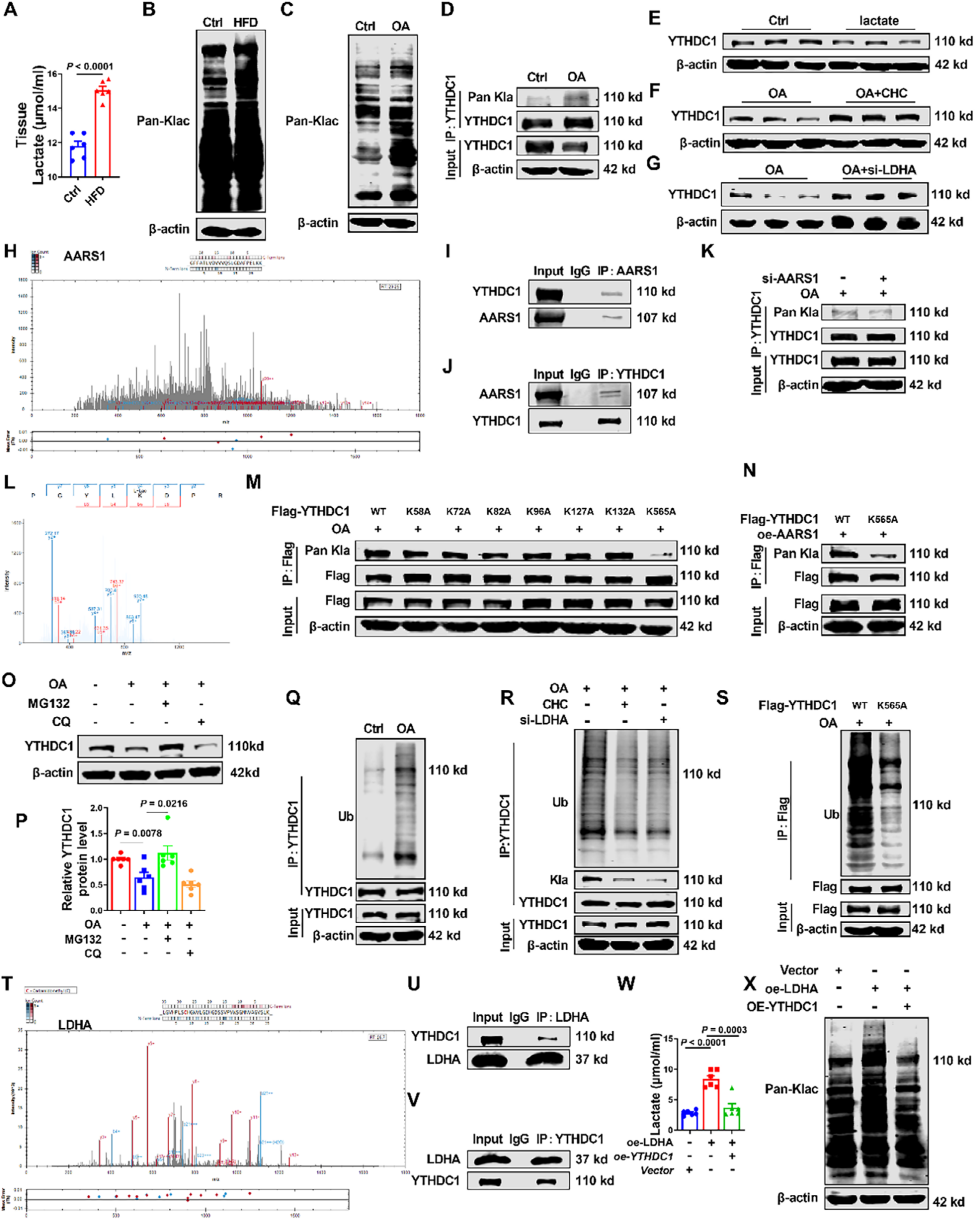
AARS1‐mediated lactylation modification drives YTHDC1 ubiquitination to inhibit its protein expression. A) Statistical data for lactate contents in mice liver. *n* = 6. B,C) Western blot analysis of Pan‐Kla in tissue and HepG2 cells. *n* = 4. D) HepG2 cell extracts were immunoprecipitated with an anti‐YTHDC1 antibody and then immunoblotted with antibodies specific for Pan‐Kla and YTHDC1. *n* = 4. E–G) Western blot analysis of YTHDC1 protein levels in different groups. *n* = 6. H) Secondary spectrum of LC‐MS analysis. I,J) HepG2 cell extracts were immunoprecipitated with anti‐YTHDC1 and anti‐AARS1 antibodies and then immunoblotted with antibodies specific for YTHDC1 and AARS1. *n* = 4. K) HepG2 cell extracts were immunoprecipitated with anti‐YTHDC1 antibody and then immunoblotted with antibodies specific for Pan‐Kla and YTHDC1. *n* = 4. L) Secondary spectrum of l‐lactylation modification omics analysis. M,N) HepG2 cell extracts were immunoprecipitated with anti‐Flag antibody and then immunoblotted with antibodies specific for Pan‐Kla and Flag. *n* = 4. O,P) Western blot analysis of YTHDC1 protein levels in the Ctrl, OA, OA+MG132, and OA+CQ groups. *n* = 6. Q–S) HepG2 cell extracts were immunoprecipitated with anti‐YTHDC1 and anti‐Flag antibody and then immunoblotted with antibodies specific for Ub, Flag, and YTHDC1. *n* = 4. T) Secondary spectrum of LC‐MS analysis. U,V) HepG2 cell extracts were immunoprecipitated with anti‐YTHDC1 and anti‐LDHA antibodies and then immunoblotted with antibodies specific for YTHDC1 and LDHA. *n* = 4. W) Statistical data of lactate contents in HepG2 cells. *n* = 6. X) Western blot analysis of Pan‐Kla in HepG2 cells. *n* = 4. Data are presented as mean ± SEM.

### 2.4 Increased Lactate Accumulation and Lactylation Drive the Ubiquitination‐Mediated Degradation of YTHDC1

Previous studies have shown that hepatocytes exhibit enhanced glycolysis in MASLD, leading to excessive lactate production and accumulation. Lactate can non‐enzymatically bind to lysine residues on proteins, forming lactylation modifications.^[^ ^25^, ^26^, ^27^, ^28^ ^]^ We observed significantly elevated lactate, pyruvate and protein lactylation levels in the liver tissues of HFD‐induced mice and OA‐treated HepG2 cells (Figure 3A–C; Figures S4A and S5A,B, Supporting Information). Using co‐immunoprecipitation (Co‐IP) analyses, we observed that the lactylation level of YTHDC1 was significantly elevated in OA‐induced HepG2 cells (Figure 3D). To investigate how lactate influences YTHDC1 expression, we demonstrated that exogenous addition of lactate markedly reduced YTHDC1 protein levels (Figure 3E; Figure S4B, Supporting Information). α‐Cyano‐4‐hydroxycinnamic acid (CHC), an inhibitor of monocarboxylate transporters that facilitate the transport of lactate and pyruvate,^[^ ^29^, ^30^ ^]^ was used to modulate lactate levels. Lactate dehydrogenase A (LDHA) primarily catalyzes the conversion of pyruvate to lactate during anaerobic glycolysis, replenishing nicotinamide adenine dinucleotide (NAD^+^) by reducing pyruvate to lactate and oxidizing NADH to NAD^+.[^ ^31^, ^32^ ^]^ To reduce lactate levels, treatment with CHC or siRNA targeting LDHA (si‐LDHA) in OA‐induced HepG2 cells led to inhibited the lactylation level and increased YTHDC1 protein expression (Figure 3F,G; Figures S4C,D and S5C, Supporting Information). These findings indicate that in MASLD, elevated lactate levels and increased YTHDC1 lactylation lead to reduced YTHDC1 expression.

Through Co‐IP analyses of YTHDC1‐associated proteins, followed by liquid chromatography‐mass spectrometry (LC‐MS) analysis, we identified AARS1 as a novel binding partner of YTHDC1 (Figure 3H). Through western blot analysis, we observed elevated AARS1 expression in in MASLD patients as well as in corresponding cellular and animal models (Figure S6A,B, Supporting Information). Co‐IP experiments provided strong evidence that YTHDC1 binds to AARS1 (Figure 3I,J). To evaluate the effect of AARS1 lactylation on YTHDC1, we performed another Co‐IP experiment. In comparison with the OA group, the OA+si‐AARS1 group showed reduced lactylation levels of YTHDC1 (Figure 3K; Figure S4E,F, Supporting Information). To systematically map the lactylation sites on YTHDC1, we performed l‐lactylation modification omics analysis. Seven putative lactylation sites (K58, K72, K82, K96, K127, K132, and K565) were identified in YTHDC1 (Figure 3L; Figure S4G–L, Supporting Information). Using site‐directed mutagenesis, we observed a marked reduction in global YTHDC1 lactylation levels, specifically upon K565 substitution, whereas mutations at other candidate sites showed no significant effect (Figure 3M). Notably, AARS1 overexpression failed to modulate YTHDC1 lactylation when the K565 site was mutated, demonstrating that AARS1‐mediated lactylation of YTHDC1 strictly depends on this critical lysine residue (Figure 3N). To explore the degradation mechanism of YTHDC1, we treated the cells with MG132 (a proteasome inhibitor) and chloroquine (an autophagy inhibitor). These results suggested that lactylated YTHDC1 undergoes degradation through ubiquitination (Figure 3O,P). In OA‐induced HepG2 cells, the ubiquitination level of YTHDC1 increased, which correlated with reduced protein expression (Figure 3Q). After CHC treatment, si‐LDHA treatment, or K565 site mutation, the ubiquitination level of YTHDC1 decreased, indicating modulation of its degradation pathway (Figure 3R,S). Intriguingly, LC‐MS analysis revealed an interaction between YTHDC1 and LDHA, and Co‐IP experiments provided strong evidence that YTHDC1 binds to LDHA (Figure 3T–V).

We also found that LDHA protein expression was significantly elevated in MASLD patients as well as in corresponding cellular and animal models (Figure S5D–J, Supporting Information). In HepG2 cells, LDHA overexpression significantly increased intracellular lactate and protein lactylation levels, whereas co‐overexpression of YTHDC1 effectively reversed this metabolic alteration, indicating a regulatory role of YTHDC1 in counteracting LDHA‐driven lactate accumulation (Figure 3W,X; Figure S4M,N, Supporting Information). In addition, extending the duration of OA induction led to a decrease in the binding between LDHA and YTHDC1(Figure S5K,L, Supporting Information). Collectively, these findings demonstrate that AARS1 mediates the K565‐specific lactylation of YTHDC1 to drive its ubiquitination‐dependent degradation, thereby reducing YTHDC1 protein expression in MASLD. To investigate the role of YTHDC1 in regulating MASLD independently of lactylation, we inhibited lactate production using CHC and subsequently silenced YTHDC1. This resulted in a significant increase in cellular TC and TG levels, as well as an expansion of Oil Red O staining areas (Figure S5M–P, Supporting Information). These results suggest that YTHDC1 is capable of directly regulating MASLD. Furthermore, overexpression of YTHDC1 counteracted the OA‐induced elevation of lactate levels and global protein lactylation (Figure S5Q,R, Supporting Information). We found that exogenous lactate administration markedly reversed the protective effect of YTHDC1 overexpression (Figure S5S–V, Supporting Information). Furthermore, our data indicated the presence of a functional interplay between YTHDC1 and LDHA in orchestrating lactate production and maintaining protein lactylation balance.

### 2.5 Identification of the Targets of YTHDC1 in MASLD

To investigate the potential mechanisms underlying the effects of YTHDC1 in the progression of MASLD, we performed RNA‐seq analysis on mice that received tail vein injections of *AAV‐Vector* and *AAV‐Ythdc1* (Figure S7A, Supporting Information). Gene ontology (GO) analysis revealed that the differentially expressed genes were associated with biological processes and molecular functions related to lipid metabolism, signal transduction, immune responses, and cellular structure. The differentially expressed genes related to lipid metabolism were involved in the production, regulation, and metabolism of lipids and organic acids, and served as key players in cellular energy supply, signaling molecule synthesis, and membrane structure maintenance (Figure S7B, Supporting Information). Kyoto Encyclopedia of Genes and Genomes (KEGG) pathway analysis revealed that these differentially expressed genes were enriched in pathways related to lipid metabolism, immune responses, and cell proliferation (Figure S7C, Supporting Information). Gene set enrichment analysis (GSEA) further revealed that the genes influenced by YTHDC1 were associated with cellular responses to oxidized low‐density lipoprotein particles, fatty acid transport, spliceosome complex, Class I MHC response antigen presentation, folding assembly, and peptide loading (Figure S7D, Supporting Information). These findings suggested that YTHDC1 plays a regulatory role in the development and progression of MASLD by influencing key biological processes and pathways related to lipid metabolism and immune responses.

**Figure 4:**
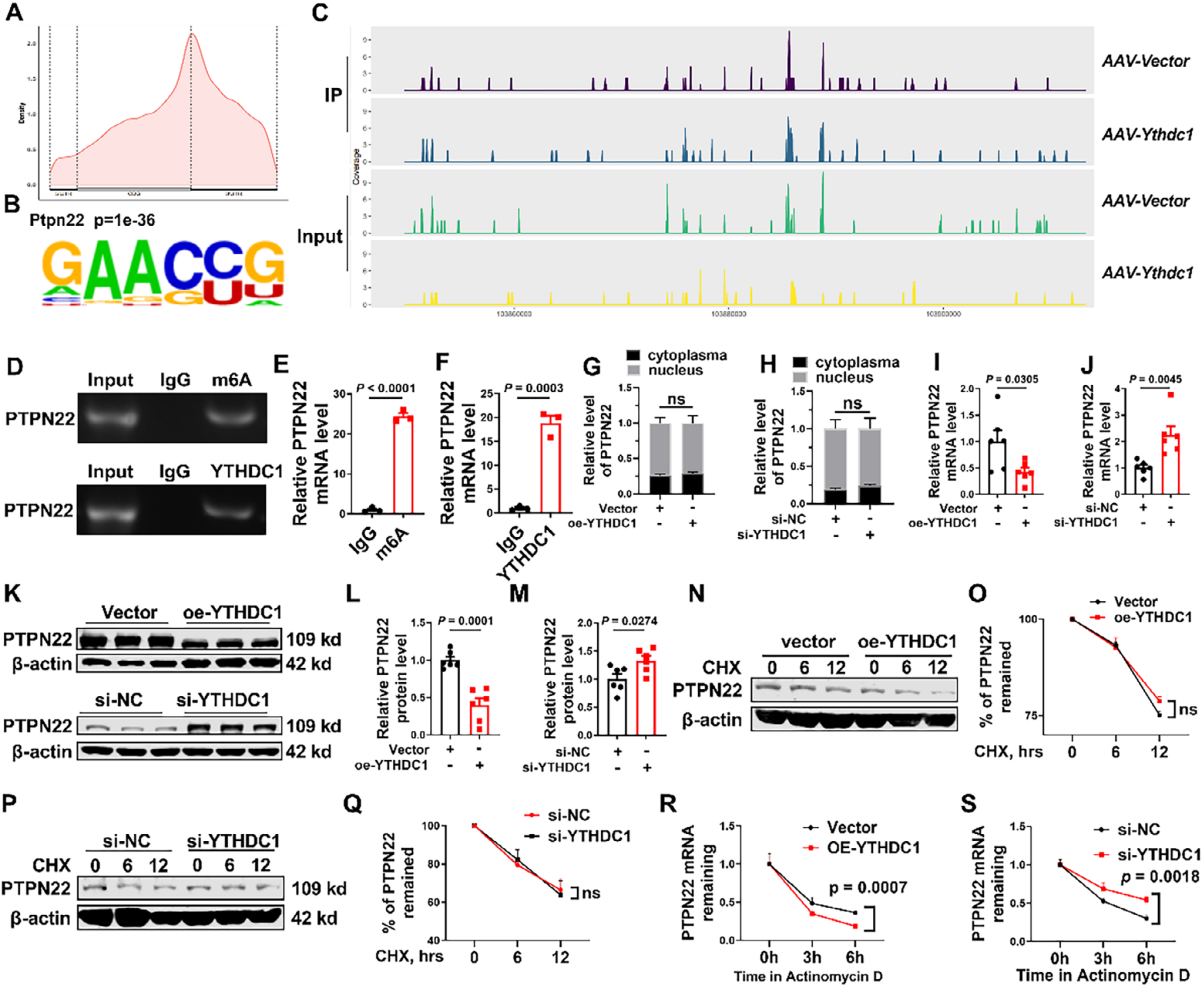
YTHDC1 targets Ptpn22 by destabilizing its mRNA. A) Density distribution analysis of binding targets to m6A. B) Representative PTPN22 potential binding motifs. C) MeRIP‐seq analysis of m6A modification sites on Ptpn22 transcripts. D–F) RIP analysis of the *PTPN22* mRNA level. *n* = 3. G) PCR analysis of *PTPN22* mRNA distribution in the nucleus and cytoplasm after overexpression of YTHDC1. *n* = 6. H) PCR analysis of the nuclear and cytoplasmic distribution of *PTPN22* mRNA after silencing YTHDC1. *n* = 6. I) PCR analysis of *PTPN22* mRNA level after overexpression of YTHDC1. *n* = 6. J) PCR analysis of *PTPN22* mRNA level after silencing YTHDC1. *n* = 6. K–M) Western blot analysis of PTPN22 protein levels after overexpression and silencing of YTHDC1. *n* = 6. N–Q) Western blot analysis of the protein level of PTPN22 induced by cycloheximide after overexpression and silencing of YTHDC1. *n* = 3. R, S) PCR analysis of actinomycin D‐induced *PTPN22* mRNA levels after overexpression and silencing of YTHDC1. *n* = 6. Data are presented as mean ± SEM.

YTHDC1, as a well‐known m6A reader, performs its function by binding to and influencing m6A‐methylated transcripts.^[^ ^7^, ^33^, ^34^, ^35^ ^]^ To investigate the methylation levels of intracellular mRNA at the transcriptome level, we performed methylated RNA immunoprecipitation combined with next‐generation sequencing (meRIP‐seq) in two groups of mice: *AAV‐Vector* and *AAV‐Ythdc1*. The analysis revealed that these m6A modifications were primarily located in protein‐coding transcripts (68.48%) and enriched near stop codons (25.28%) (**Figure** **4**A). The meRIP analysis identified 571 potential m6A‐modified targets. GO analysis showed that these targets were enriched in biological processes, such as signal transduction and regulation, including protein phosphorylation, cell behavior and function regulation, and inflammation and immune responses (Figure S7E,F, Supporting Information). Twenty‐three potential targets were identified by overlapping the differentially expressed gene sets from RNA‐seq and meRIP‐seq (Figure S7G, Supporting Information). Among these, *Ptpn22* stands out because of its involvement in inflammatory responses and protein phosphorylation. PTPN22 is a tyrosine phosphatase that plays a critical role in immune regulation by participating in T– and B cell signaling, regulating their activation, and maintaining immune tolerance. Additionally, PTPN22 affects inflammatory responses by regulating NLRP3 inflammasome activation. Using meRIP‐seq analysis, we identified the m6A binding motif of Ptpn22 with HOMER and mapped the m6A modification sites on the *Ptpn22* transcript (Figure 4B,C). These findings suggest that *Ptpn22* is a downstream target of YTHDC1. To test this hypothesis, we performed RNA immunoprecipitation (RIP) experiments. As expected, *Ptpn22* bound to both m6A and YTHDC1 (Figure 4D–F). These results demonstrate that YTHDC1 recognizes m6A modification sites on *Ptpn22*, suggesting that *Ptpn22* is a downstream target of YTHDC1 and participates in the progression of MASLD.

### 2.6 YTHDC1 Suppresses the Expression of PTPN22 by Disrupting mRNA Stability

YTHDC1 plays a crucial role in the nuclear export of mRNAs modified with m6A. YTHDC1 interacts with the splicing factor SRSF3 to mediate the transport of these mRNAs from the nucleus to the cytoplasm. When YTHDC1 is knocked out, m^6^A‐modified mRNAs accumulate in the nucleus, whereas their levels in the cytoplasm decrease, indicating that YTHDC1 is essential for proper nuclear clearance and cytoplasmic distribution of m^6^A‐modified transcripts.^[^ ^36^, ^37^ ^]^ To investigate the role of YTHDC1 in the regulation of *Ptpn22* expression, we first separately extracted RNA from the nucleus and cytoplasm. The PCR results showed that neither overexpression nor silencing of YTHDC1 affected the distribution of PTPN22 mRNA between the nucleus and cytoplasm (Figure 4G,H). Next, we examined the expression of *Ptpn22* at both the transcriptional and protein levels after modulating YTHDC1 expression. The PCR results revealed that overexpression of YTHDC1 significantly reduced the mRNA and protein levels of *Ptpn22*, whereas silencing of YTHDC1 led to a significant increase in *Ptpn22* mRNA and protein levels (Figure 4I–M). To explore the mechanisms underlying these changes, we used cycloheximide to inhibit protein synthesis. This analysis revealed that neither overexpression nor silencing of YTHDC1 affected the stability of the *Ptpn22* protein (Figure 4N–Q). However, when transcription was inhibited using actinomycin D, overexpression of YTHDC1 reduced the stability of *Ptpn22* mRNA, whereas silencing of YTHDC1 increased its stability. These findings suggested that YTHDC1 binds to *Ptpn22* mRNA and regulates its stability, thereby influencing Ptpn22 expression (Figure 4R,S). Through these experiments, we demonstrated that YTHDC1 suppressed the expression of *Ptpn22* by destabilizing its mRNA, thereby alleviating the progression of MASLD.

### 2.7 Modulating PTPN22 Expression Influences the Progression of MASLD

**Figure 5:**
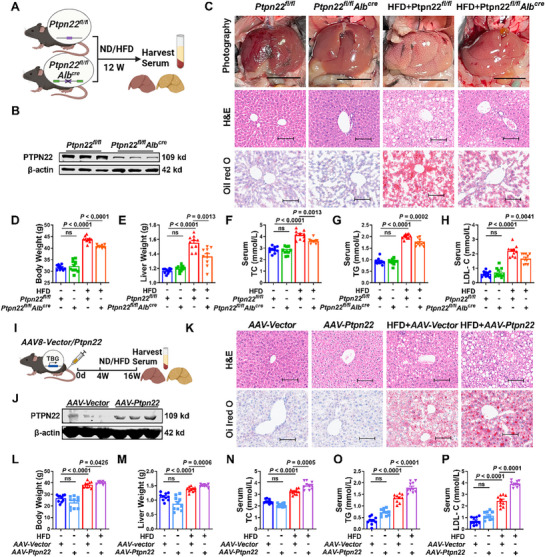
Modulating PTPN22 expression regulates the progression of MASLD. A) Schematic diagram showing liver‐specific knockout of PTPN22 mice. B) Western blot analysis of PTPN22 levels in *Ptpn22^fl/fl^* mice and *Ptpn22^fl/fl^Alb^cre^* mice. *n* = 3. C) Representative images of livers and liver sections stained with HE and Oil Red O after HFD feeding in *Ptpn22^fl/fl^* mice and *Ptpn22^fl/fl^Alb^cre^* mice. Scale bars=100 µm. *n* = 4. D,E) Liver weight and body weight of *Ptpn22^fl/fl^* mice and *Ptpn22^fl/fl^Alb^cre^* mice after HFD induction. *n* = 10. F–H) After 12 weeks of HFD induction, the TC, TG and LDL‐C levels in serum of *Ptpn22^fl/fl^* mice and *Ptpn22^fl/fl^Alb^cre^* mice. *n* = 10. I) Schematic diagram of the process of tail vein injection of *AAV‐Ptpn22* expressing PTPN22 in mice. J) Western blot analysis of PTPN22 protein level in the liver of AAV‐infected mice. *n* = 3. K) Representative images of liver sections of AAV‐infected mice stained with HE and Oil Red O. Scale bars=100 µm. *n* = 4. L,M) The liver weight and body weight of AAV‐infected mice. *n* = 10. N–P) After 12 weeks of HFD induction, the TC, TG and LDL‐C levels in serum of AAV‐infected mice. *n* = 10. Data are presented as mean ± SEM.

In previous experiments, we found that YTHDC1 was underexpressed in patients with MASLD, HFD‐fed mice, and OA‐induced HepG2 and Thle‐2 cells. To further investigate the regulation of *Ptpn22* by YTHDC1, we examined the expression levels of *Ptpn22* in these models. PCR and western blot analyses showed that *Ptpn22* expression was significantly increased in patients with MASLD, HFD‐fed mice, and OA‐induced HepG2 and Thle‐2 cells (Figure S8A–F, Supporting Information). To explore the regulatory role of PTPN22 in the progression of MASLD, we generated liver‐specific conditional knockout mice for PTPN22 (**Figure** **5**A). In comparison with *Ptpn22^fl/fl^* mice, *Ptpn22^fl/fl^Alb^cre^* mice exhibited significantly reduced mRNA and protein expression levels of PTPN22 (Figure 5B; Figure S8G,H, Supporting Information). Under HFD conditions, the livers of *Ptpn22^fl/fl^Alb^cre^* mice appeared smaller and more reddish than those of *Ptpn22^fl/fl^* mice. H&E staining revealed fewer lipid droplets in hepatocytes and a reduced degree of inflammatory cell infiltration in *Ptpn22^fl/fl^Alb^cre^* mice. Oil Red O staining revealed a significant reduction in lipid droplets within the hepatocytes. Overall, the pathological changes in *Ptpn22^fl/fl^Alb^cre^* mice were less severe than those in *Ptpn22^fl/fl^* mice (Figure 5C; Figure S8I, Supporting Information). As in previous experiments, HFD feeding significantly increased the liver and body weights of *Ptpn22^fl/fl^* mice. However, these increments were mitigated in *Ptpn22^fl/fl^Alb^cre^* mice, which exhibited reduced body and liver weights (Figure 5D,E). Additionally, PTPN22 knockout counteracted the HFD‐induced elevation of TC and TG levels in both tissues and serum (Figure 5F,G; Figure S8J,K, Supporting Information). After 12 weeks of HFD feeding, the serum levels of ALT, AST, and LDL‐C were significantly lower in *Ptpn22^fl/fl^Alb^cre^* mice than in *Ptpn22^fl/fl^* mice (Figure 5H; Figure S8L,M, Supporting Information). ALT and AST levels in the liver tissues followed the same trend (Figure S8N,O, Supporting Information). These findings suggest that PTPN22 plays a critical role in the progression of MASLD, and that its liver‐specific knockout alleviates HFD‐induced pathological changes, including lipid accumulation and liver damage.

To further elucidate the role of PTPN22 in the progression of MASLD, we overexpressed PTPN22 in the mouse liver through intravenous injection of an AAV expressing PTPN22 (*AAV‐Ptpn22*) (Figure 5I). 4 weeks post‐infection, the efficiency of AAV‐mediated overexpression was assessed using western blotting and reverse transcription (RT)‐PCR. In comparison with *AAV‐Vector* mice, *Ptpn22* protein and mRNA expression levels were significantly elevated in *AAV‐Ptpn22* mice (Figure 5J; Figure S8P,Q, Supporting Information). Gross imaging revealed that overexpression of PTPN22 exacerbated the pale appearance and liver enlargement induced by HFD feeding. H&E staining demonstrated increased lipid droplet deposition, more severe ballooning degeneration, and greater inflammatory cell infiltration in the livers of *AAV‐Ptpn22* mice than in *AAV‐Vector* controls. Similarly, Oil Red O staining revealed significantly increased lipid droplet deposition in the liver tissues of *AAV‐Ptpn22* mice (Figure 5K; Figure S8R, Supporting Information). After HFD treatment, the liver and body weights significantly increased in *AAV‐Vector* mice, with even greater increments observed in *AAV‐Ptpn22* mice (Figure 5L,M). Furthermore, after 12 weeks of HFD feeding, *AAV‐Ptpn22* mice exhibited significantly elevated serum and tissue levels of TC, TG, ALT, AST, and LDL‐C than *AAV‐Vector* mice (Figure 5N–P; Figure S8S–X, Supporting Information). These findings suggest that under HFD conditions, silencing PTPN22 expression alleviates the progression of MASLD, whereas liver‐specific overexpression of PTPN22 exacerbates lipid accumulation and liver damage.

Additionally, we conducted functional validation of PTPN22 in HepG2 and Thle‐2 cells. First, we constructed a plasmid vector carrying oe‐PTPN22. In comparison with vector transfection, overexpression of PTPN22 resulted in significantly increased protein and mRNA levels in HepG2 and Thle‐2 cells (Figure S9A,B,M,N, Supporting Information). In both HepG2 and Thle‐2 cells, TC and TG levels were significantly elevated in the OA‐treated group in comparison with those in the vector group. Overexpression of PTPN22 further exacerbated this increase in TC and TG levels (Figure S9C,D,O,P, Supporting Information). Oil Red O staining revealed that after OA stimulation, upregulation of PTPN22 significantly aggravated lipid deposition in HepG2 and Thle‐2 cells in comparison with the vector group (Figure S9E,F,Q,R, Supporting Information). These findings demonstrated that PTPN22 overexpression exacerbates lipid accumulation and promotes the pathological features of MASLD in vitro.

Next, we constructed siRNAs targeting PTPN22. Western blot and RT‐PCR analyses confirmed that the transfection of HepG2 cells with si‐PTPN22 significantly reduced both the protein and mRNA expression levels of PTPN22 (Figure S9G,H,S,T, Supporting Information). After OA exposure, the total cholesterol (TC) and triglyceride (TG) levels in the si‐PTPN22 group were significantly lower than those in the si‐NC group (Figure S9I,J,U,V, Supporting Information). Oil Red O staining showed that si‐PTPN22 alleviated OA‐induced lipid deposition in HepG2 cells (Figure S9K,L,W,X, Supporting Information). Overall, these experiments revealed that upregulation of PTPN22 significantly exacerbated lipid accumulation in HepG2 and Thle‐2 cells, whereas the inhibition of PTPN22 expression effectively reduced excessive lipid accumulation.

### 2.8 PTPN22 Affects Lipid Accumulation in Hepatocytes by Modulating the Phosphorylation Modification of Tyrosine 861 in NLRP3

**Figure 6:**
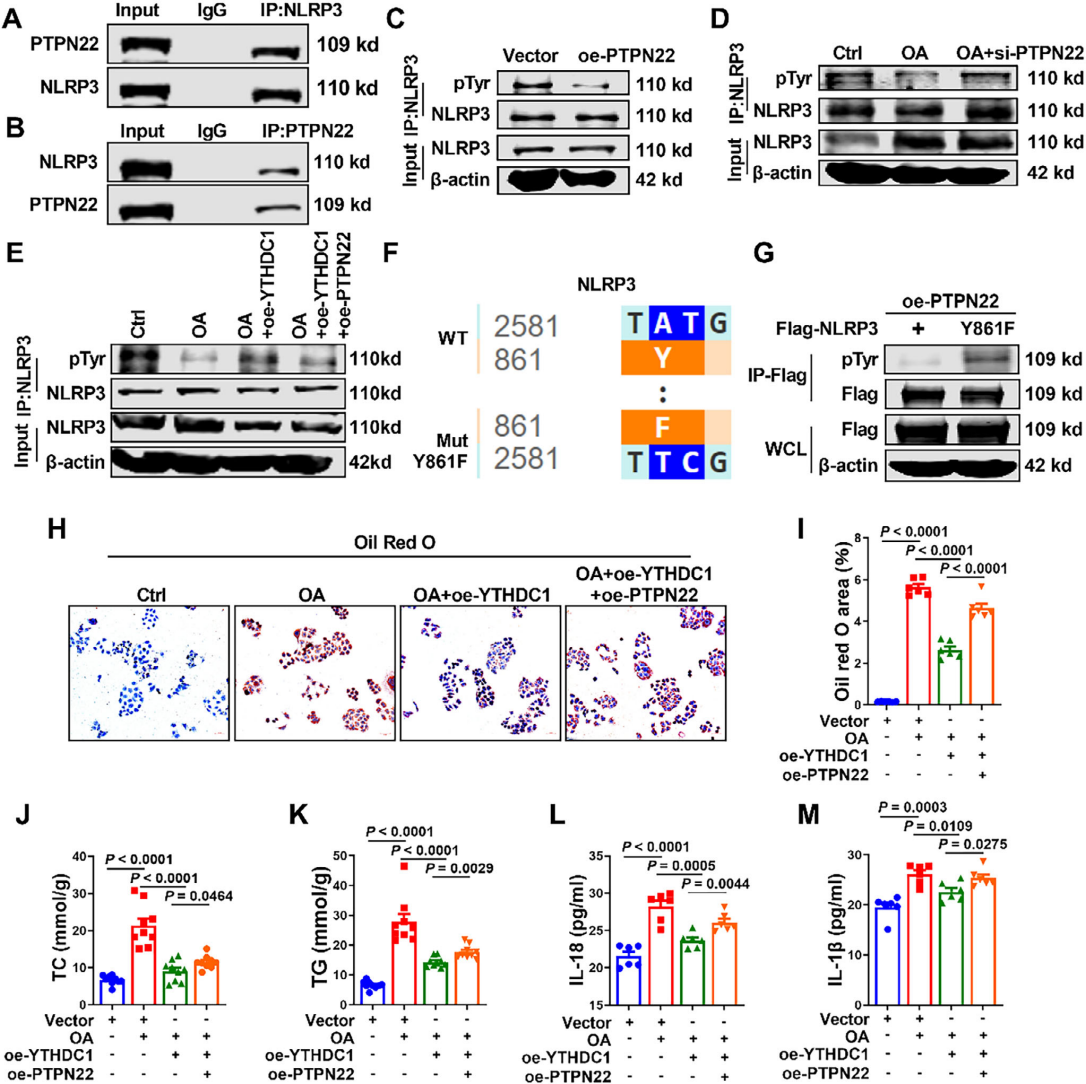
PTPN22 affects lipid accumulation in hepatocytes by modulating the phosphorylation of tyrosine 861 in NLRP3. A,B) HepG2 cell extracts were immunoprecipitated with anti‐NLRP3 and anti‐PTPN22 antibodies and then immunoblotted with antibodies specific for NLRP3 and PTPN22. *n* = 4. C–E) HepG2 cells extracts were immunoprecipitated with anti‐NLRP3 antibody and then immunoblotted with antibodies specific for pTyr and NLRP3. *n* = 4. F) A schematic diagram illustrating the mutation of lysine at position 861 of NLRP3. G) FLAG‐NLRP3 and FLAG‐NLRP3^Y861F^ were transfected into HepG2 cells. Whole‐cell extracts were immunoprecipitated with anti‐FLAG antibodies and then immunoblotted with antibodies specific for pTyr and FLAG. *n* = 4. H) Oil Red O staining of HepG2 cell coverslips. Scale bars=50 µm. *n* = 6. I) Statistical data for Oil red O staining. *n* = 6. J,K) Statistical data of TC and TG in HepG2 cells. *n* = 9. L,M) Quantitative data of IL‐1β and IL‐18 contents in HepG2 cells. *n* = 6. Data are presented as mean ± SEM.

NLRP3 plays an important role in MASLD, particularly in the inflammatory response triggered by lipid accumulation. Activation of the NLRP3 inflammasome promotes the secretion of pro‐inflammatory cytokines such as IL‐1β and IL‐18, exacerbating liver inflammation and fibrosis.^[^ ^38^, ^39^ ^]^ Co‐IP experiments provided strong evidence that PTPN22 binds to NLRP3 (**Figure** **6**A,B). To evaluate the effect of PTPN22 dephosphorylation on NLRP3 expression, we performed another Co‐IP experiment. In comparison with the vector group, the phosphorylation level of NLRP3 was reduced in the oe‐PTPN22 group (Figure 6C). Similarly, after OA induction, the phosphorylation level of NLRP3 was reduced, which was reversed by silencing PTPN22, resulting in an increase in NLRP3 phosphorylation (Figure 6D). Subsequent experiments revealed that under OA induction, overexpression of YTHDC1 increased the phosphorylation level of NLRP3. To confirm whether YTHDC1 affects NLRP3 phosphorylation through PTPN22, we co‐overexpressed PTPN22 and YTHDC1. Co‐IP analysis revealed that PTPN22 overexpression counteracted the YTHDC1‐induced increase in NLRP3 phosphorylation (Figure 6E). Using UniProt and NetPhos‐3.1, we identified tyrosine residue 861 of NLRP3 as a dephosphorylation target site for PTPN22. To verify this, we constructed a FLAG‐tagged NLRP3 overexpression plasmid and mutated the tyrosine residue at position 861 to phenylalanine (NLRP3Y861F) (Figure 6F). In HepG2 cells, FLAG‐NLRP3 or FLAG‐NLRP3Y861F were co‐expressed with PTPN22. Co‐IP experiments revealed that the dephosphorylation effect of PTPN22 on NLRP3 was abolished when tyrosine 861 was mutated to phenylalanine (Figure 6G). Next, we investigated the effects of NLRP3 phosphorylation on lipid accumulation. Oil Red O staining showed that under OA stimulation, overexpression of YTHDC1 inhibited lipid accumulation in HepG2 cells; however, this inhibitory effect was reversed by further overexpression of PTPN22 (Figure 6H,I). Similarly, the TC and TG levels were elevated in HepG2 cells after OA treatment. Overexpression of YTHDC1 reduced TC and TG levels, and additional overexpression of PTPN22 and YTHDC1 also reduced the TC and TG levels; however, further overexpression of PTPN22 significantly increased these levels in comparison with those observed in the YTHDC1‐overexpression group (Figure 6J,K). Given the role of NLRP3 in inflammation, we measured the levels of IL‐1β and IL‐18. In comparison with the Vector group, the OA group showed elevated IL‐1β and IL‐18 levels. Overexpression of YTHDC1 inhibited the production of these inflammasome‐associated cytokines; however, this effect was reversed by the overexpression of PTPN22 (Figure 6L,M). On the basis of these results, we conclude that YTHDC1 influences the phosphorylation level of NLRP3 through PTPN22. Specifically, PTPN22 mediates the dephosphorylation of tyrosine 861 on NLRP3, thereby regulating its activation and downstream effects.

### 2.9 Mebendazole Improves MASLD by Acting on YTHDC1

**Figure 7:**
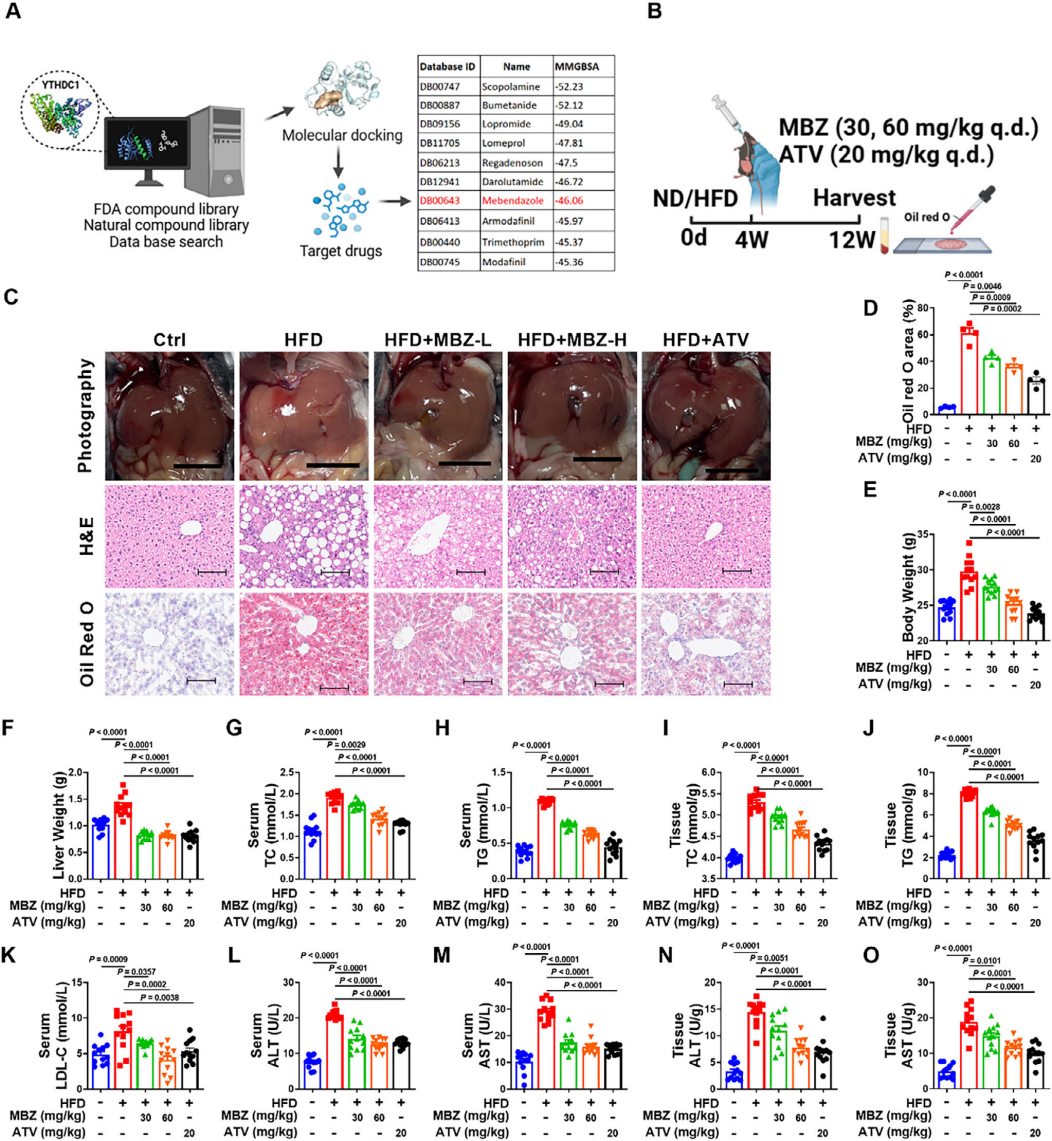
Mebendazole improves the progression of HFD‐induced MASLD in mice. A) Virtual screening was performed using 19 377 chemical small molecules from the Taosu Natural Product Library and the drug database of 2452 FDA‐approved molecules prior to 2024. B) Diagram illustrating the experimental design and procedures with mice. MBZ: Mebendazole C) Representative images showing morphological changes in the liver. For histopathological studies, liver tissue sections were stained with H&E and Oil red O. Scale bars=100 µm. *n* = 4. D) Statistical data for Oil red O staining. *n* = 4. E,F) Body and liver weights in the indicated groups (Ctrl, HFD, HFD+MBZ‐L, HFD+MBZ‐H, HFD+ATV). *n* = 12. G–O) Statistical data of TC, TG, LDL‐C, ALT, and AST levels in serum or tissue samples. *n* = 12. Data are presented as mean ± SEM.

On the basis of the protein structure of YTHDC1, we conducted virtual screening of two libraries: the FDA‐approved drug database, which contained 2452 molecules, and the Taosu Natural Product Library, with 19 377 chemical small molecules. From this analysis, mebendazole was identified as the most promising drug candidate (**Figure** **7**A). The direct interaction between mebendazole and YTHDC1 was further verified by temperature‐dependent cellular thermal shift assay (CETSA) and Drug Affinity Responsive Target Stability (DARTs) (Figure S10A,B, Supporting Information). Previous studies have demonstrated that atorvastatin effectively improves MASLD by enhancing lipid metabolism, inhibiting inflammation, and regulating antioxidant and redox reactions.^[^ ^40^, ^41^ ^]^ After 12 weeks of HFD induction and 8 weeks of drug treatment, tissue samples were collected from mice (Figure 7B). Gross photography revealed that both mebendazole and atorvastatin significantly improved the pale appearance and enlarged liver size caused by HFD feeding. H&E staining revealed that treatment with mebendazole and atorvastatin significantly reduced lipid droplet accumulation, inhibited inflammatory responses, and alleviated hepatocyte swelling. Similarly, Oil Red O staining showed marked inhibition of lipid accumulation in the liver following treatment with mebendazole and atorvastatin (Figure 7C,D). Treatment with mebendazole and atorvastatin also significantly reduced the body and liver weights of the HFD‐fed mice (Figure 7E,F). Serum and tissue analyses revealed that both drugs effectively lowered TC and TG levels (Figure 7G–J). Serum LDL‐C levels showed a similar reduction with mebendazole and atorvastatin treatments (Figure 7K). Additionally, treatment with mebendazole and atorvastatin significantly reduced ALT and AST levels in serum and tissues, mitigating HFD‐induced liver damage (Figure 7L–O). For examining the regulatory effects of mebendazole on the YTHDC1 pathway, western blot showed that mebendazole markedly increased YTHDC1 protein expression, while significantly suppressing the protein levels of PTPN22 and NLRP3 (Figure S10C–E, Supporting Information). Furthermore, we administered mebendazole to hepatocyte‐specific YTHDC1 knockout mice. H&E and Oil Red O staining demonstrated that mebendazole treatment lost its therapeutic effects on lipid accumulation and inflammatory cell infiltration upon YTHDC1 knockout (Figure S10F, Supporting Information). Consistent with these findings, measurements of TC, TG, AST, and ALT in serum and liver homogenates yielded concordant results (Figure S10G–L, Supporting Information). These data confirm that mebendazole exerts its therapeutic effects against MASLD in a YTHDC1‐dependent manner. In summary, both mebendazole and atorvastatin demonstrated substantial therapeutic potential for reducing HFD‐induced liver damage and lipid accumulation in a mouse model of MASLD. This therapeutic effect is likely mediated by the modulation of YTHDC1.

## 3 Discussion

**Figure 8:**
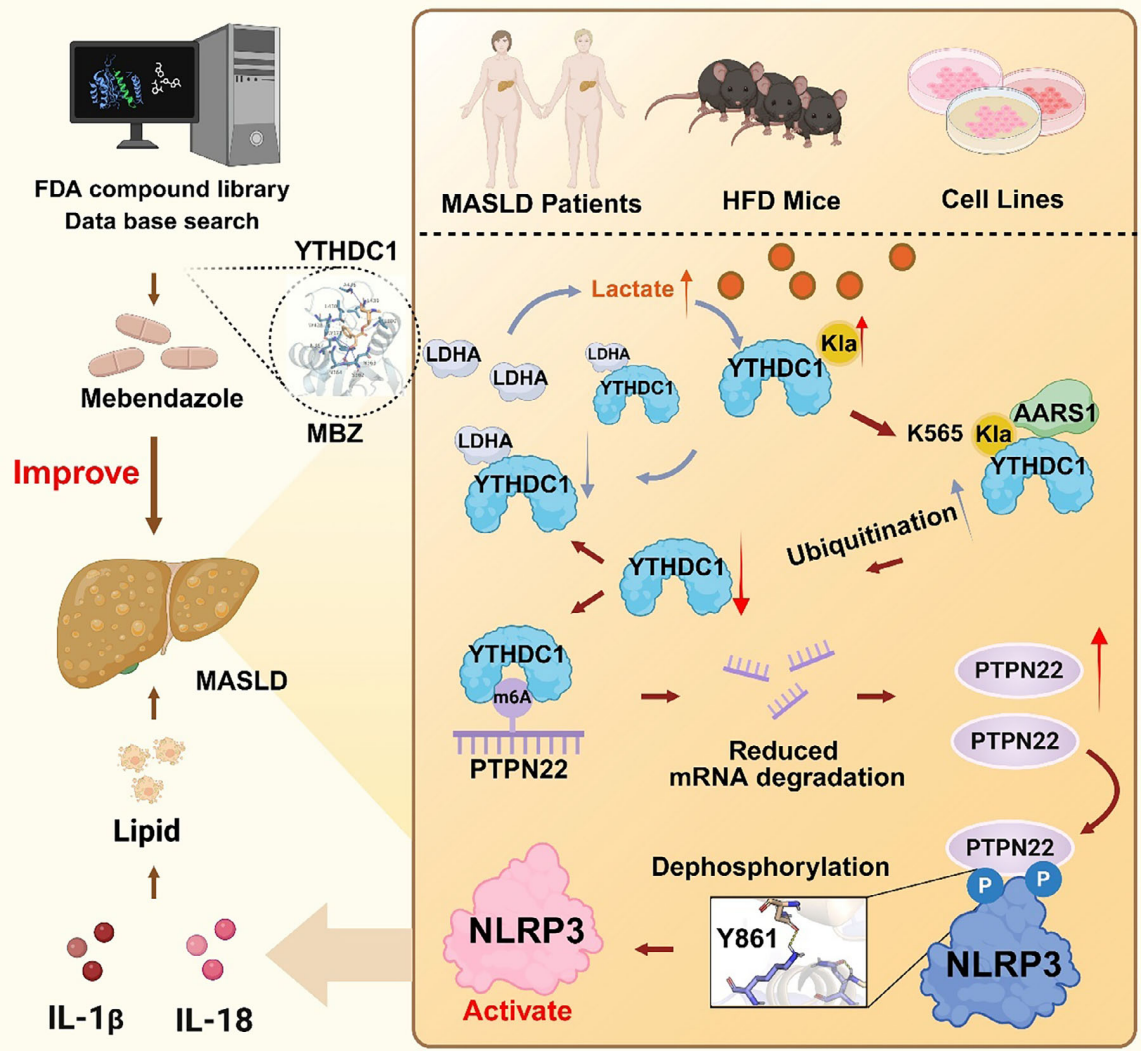
Graphical Abstract. In MASLD, the lactylation and ubiquitination levels of YTHDC1 are increased, leading to a reduction in its protein expression. AARS1 regulates the lactylation of YTHDC1 by targeting its K565 site. Under MASLD conditions, diminished YTHDC1‐LDHA binding elevates free LDHA levels, which enhances YTHDC1 lactylation to suppress its expression. The decreased YTHDC1 levels result in reduced degradation of PTPN22 mRNA, thereby enhancing its stability. As a tyrosine phosphatase, PTPN22 binds to NLRP3 at lysine 861, inhibiting NLRP3 phosphorylation, which promotes the activation of NLRP3 and drives inflammation and lipid accumulation. Mebendazole may treat MASLD by regulating YTHDC1.

Our study showed that increased lactylation of YTHDC1 enhanced the ubiquitination of YTHDC1 and reduced its expression in MASLD. YTHDC1 recognizes the m6A modification sites of PTPN22 and destabilizes its mRNA. PTPN22 dephosphorylates NLRP3 at tyrosine 861, aggravating IL‐1β and IL‐18 release and lipid accumulation. Additionally, through binding affinity screening, we identified mebendazole as a potential therapeutic agent for MASLD that modulates YTHDC1 expression (**Figure** **8**). This study yielded the following pivotal findings: (1) This is the first study to identify the critical role of YTHDC1 in MASLD and further elucidate the regulatory effects of lactylation modification on non‐histone proteins. (2) The findings revealed the regulatory role of YTHDC1 in mRNA stability. (3) The results provide a breakthrough by reporting the role of PTPN22 as a tyrosine phosphatase in the dephosphorylation of NLRP3 in MASLD. (4) Through screening of the FDA drug library, we identified the therapeutic potential of mebendazole for MASLD.

YTHDC1 is the primary nuclear m^6^A reader that controls the fate of m^6^A‐modified transcripts during various stages of the mRNA life cycle, including splicing, transcription, and nuclear export.^[^ ^7^, ^36^, ^42^ ^]^ Multiple studies have demonstrated that m^6^A, an important RNA modification, plays a significant regulatory role in MASLD by influencing fatty acid metabolism, inflammatory responses, and lipid metabolism.^[^ ^43^, ^44^, ^45^ ^]^ In this study, we identified, for the first time, the critical role of the m^6^A reader YTHDC1 in MASLD. Our results revealed that YTHDC1 expression was significantly reduced in MASLD and that its overexpression effectively inhibited lipid accumulation.

The YTH domain family proteins (YTHDF1‐3 and YTHDC1‐2) are a class of reader proteins that selectively recognize m6A sites on mRNA. Among these, YTHDC1 is unique for its sequence selectivity, exhibiting higher affinity for the GA* motif (guanine at the ‐1 position preceding methylated adenosine) than for the AA* motif.^[^ ^46^, ^47^ ^]^ Furthermore, YTHDC1 is the only m6A reader that localizes exclusively to the nucleus. To explore the role of YTHDC1 in m6A and gene regulation, we identified YTHDC1 targets through combined RIP‐seq and RNA‐seq analyses. On the basis of these analyses, PTPN22 emerged as a key target. Previous studies have highlighted the role of YTHDC1 in the nuclear import, splicing, and phase separation of mRNAs to form nuclear condensates.^[^ ^14^, ^48^, ^49^ ^]^ Based on previously reported functions of YTHDC1 as an m6A reader, it is known to influence the nucleocytoplasmic localization of downstream target mRNA transcripts. In our study, we initially considered this function of YTHDC1 in regulating mRNA localization. However, we found that YTHDC1 does not affect the nucleocytoplasmic localization of PTPN22 mRNA but instead influences its RNA stability. The specific mechanism remains unexplored in our research. We hypothesize that YTHDC1 may bind to specific regions of PTPN22 mRNA and trigger its degradation. Through literature review, we noted that YTHDF2, another m6A reader, plays a dual role in B‐cell malignancies both recruiting PABPC1 and recognizing m6A‐modified mRNAs to mediate their degradation.^[^ ^50^ ^]^ Unlike YTHDF2, which was previously thought to primarily regulate mRNA stability, this finding suggests that m6A readers may possess diverse regulatory roles. However, in the present study, we observed that YTHDC1 regulates the stability of PTPN22 mRNA, thereby influencing its protein expression levels. Although we observed the effect of YTHDC1 on PTPN22 mRNA stability and expression, the broader implications of the impact of YTHDC1 on other mRNAs and the specific molecular mechanisms remain unexplored. Future studies should aim to investigate these aspects to provide a comprehensive understanding of the role of YTHDC1 in MASLD and its potential as a therapeutic target.

Lactylation regulates protein expression through various mechanisms. In the tumor microenvironment, lactate accumulation induces methyltransferase‐like 3 (METTL3) upregulation in tumor‐infiltrating myeloid cells by enhancing lactylation at histone H3K18.^[^ ^51^ ^]^ In gastric cancer, high Cu levels promote the lactylation of methyltransferase‐like 16 (METTL16) at K229, which is mediated by SIRT2, without affecting METTL16 expression. Lactylated METTL16 upregulates FDX1 mRNA and protein expression through m6A modification.^[^ ^52^ ^]^ Hypoxia increases lactylation levels in microglia, boosting FGF2 expression and retinal neovascularization. The transcription factor Yin Yang‐1 (YY1) undergoes lactylation at K183 and is regulated by p300. Hyperlactylated YY1 enhances FGF2 transcription and angiogenesis, while its protein expression remains unchanged.^[^ ^53^ ^]^ Lactylation at the K91 site of transcription factor EB (TFEB) prevents its interaction with the E3 ubiquitin ligase WWP2, thereby inhibiting TFEB ubiquitination. Since ubiquitination typically leads to TFEB degradation, lactylation stabilizes TFEB by blocking ubiquitination, thereby enhancing the activity of TFEB and promoting autophagy.^[^ ^54^ ^]^ Our study revealed that AARS1 drives the ubiquitination‐mediated degradation of YTHDC1 by elevating its lactylation levels at the K565 site, ultimately suppressing YTHDC1 protein expression. AARS1 acts as an intracellular lactate sensor and lactyltransferase, binds l‐lactate with high affinity, and catalyzes adenosine triphosphate (ATP)‐dependent lysine lactylation through a lactate‐adenosine monophosphate (AMP) intermediate. AARS1 drives immune evasion and tumorigenesis by coupling lactate metabolism with proteomic reprogramming. The evolutionarily conserved AARS1 expands the non‐canonical roles of aminoacyl‐tRNA synthetases by linking metabolism to post‐translational regulation.^[^ ^55^, ^56^ ^]^ In future studies, we plan to explore the relationship between lactylation and ubiquitination.

Intriguingly, our study revealed that LDHA binds to YTHDC1 and drives lactate accumulation along with global protein lactylation. These effects were counteracted by YTHDC1 overexpression. Based on these findings, we propose that a positive feedback loop regulates the lactylation modification of YTHDC1. Specifically, following the onset of MASLD, the expression of LDHA is up‐regulated, leading to increased lactate accumulation. This promotes AARS1‐mediated lactylation modification of YTHDC1, which in turn reduces its protein expression. The downregulation of YTHDC1 further diminishes its binding to LDHA, resulting in more free LDHA that enhances lactate production and amplifies YTHDC1 lactylation, thereby forming a self‐reinforcing regulatory cycle. Wei et al. demonstrated that in pancreatic ductal adenocarcinoma (PDAC), TTK and BUB1B drive glycolytic flux by upregulating P300 expression. Mechanistically, TTK phosphorylates and activates LDHA, increasing lactate production and histone H3K18 lactylation. These interactions establish a self‐reinforcing glycolytic‐H3K18la‐TTK/BUB1B loop that exacerbates metabolic reprogramming and tumor progression in PDAC.^[^ ^57^ ^]^ In future studies, we will delve deeper into the mechanistic contributions of this positive feedback loop to MASLD progression.

In previous studies, PTPN22 has been identified as a protein tyrosine phosphatase that plays a critical role in the immune system. It participates in the regulation of T cell receptor (TCR) and B cell receptor (BCR) signaling pathways. Additionally, PTPN22 has been implicated in platelet function and arterial thrombosis.^[^ ^58^, ^59^ ^]^ In addition, the precise regulation of gene expression is governed by the dynamic equilibrium between kinase and phosphatase activities during transcriptional processes.^[^ ^60^ ^]^ In this study, we first validated the interaction between PTPN22 and NLRP3 in patients with MASLD. We confirmed the regulatory role of PTPN22 on NLRP3 and identified the specific phosphorylation sites in NLRP3 that are regulated by PTPN22. As a phosphatase, liver‐specific knockout of PTPN22 effectively counteracted HFD‐induced hepatic lipid accumulation, whereas PTPN22 overexpression exacerbated HFD‐induced lipid deposition. These findings revealed an unexpected but significant role of PTPN22 in MASLD, highlighting its potential as a promising therapeutic target for the treatment of this condition.

Development of new drugs is a difficult, long‐term, and costly process. Therefore, repurposing approved drugs for new indications is an attractive strategy to expand treatment options for patients.^[^ ^61^ ^]^ Since these drugs are already used in routine clinical practice for other purposes, they can bypass early‐stage clinical trials, thus saving time and money. The potential for drug repurposing spans all areas of medicine, with notable activity in oncology, neurology, psychiatry, and infectious diseases. Several drugs have already been repurposed for new indications, such as propranolol for treating infantile hemangiomas, thalidomide for multiple myeloma, and topiramate for migraine prevention.^[^ ^62^ ^]^ Glatiramer acetate is a synthetic random copolymer composed of four amino acids that has been used for many years to treat multiple sclerosis. Recent studies have demonstrated that in an acute myocardial ischemia mouse model, glatiramer acetate treatment exerted a beneficial, multifaceted effect on heart injury, leading to improved cardiac function and reduced scar tissue formation.^[^ ^63^ ^]^ Monoamine oxidase A (MAO‐A) catalyzes the oxidative deamination of monoamine compounds. MAO‐A is located in the outer mitochondrial membrane and plays a critical role in the central nervous system and nerve terminal tissues. Currently, MAO‐A inhibitors are essential for treating depression, Parkinson disease, and Alzheimer's disease. Recent studies have shown that MAO‐A inhibitors may also have significant effects outside the brain, including tumor immunity.^[^ ^64^ ^]^ On the basis of the protein structure of YTHDC1, we screened the FDA‐approved drug library. Among the top ten FDA‐approved drugs with strong binding ability to YTHDC1, we ultimately selected mebendazole, considering the known indications and adverse reactions associated with this drug. Mebendazole, a well‐established anthelmintic agent, is characterized by its broad‐spectrum activity and safety profile, as demonstrated over years of clinical use.^[^ ^65^ ^]^ Additionally, numerous studies have shown that mebendazole inhibits tumor progression and metastasis.^[^ ^59^, ^66^, ^67^ ^]^ Furthermore, mebendazole has been reported to significantly improve inflammatory and fibrosis‐related diseases.^[^ ^68^, ^69^ ^]^ In our study, mebendazole was identified as a candidate drug because of its binding affinity for YTHDC1. Molecular docking analysis revealed a potential interaction between mebendazole and YTHDC1. Through oral gavage, mebendazole was found to significantly ameliorate the progression of MASLD, reducing lipid accumulation and suppressing inflammatory responses.

In summary, this study demonstrated that YTHDC1 regulates the expression of PTPN22 by modulating the stability of its mRNA. PTPN22, in turn, influences the phosphorylation of NLRP3, thereby regulating inflammasome activation and mitigating the progression of MASLD. Additionally, we found that lactylation drives the expression of YTHDC1. The results also suggested that mebendazole may target YTHDC1 to treat MASLD. These findings provide novel insights into the molecular mechanisms underlying MASLD, and highlight potential therapeutic targets.

## 4 Experimental Section

### Animals and Treatments

All animal experimental protocols were approved by the Ethics Committee of the College of Pharmacy, Harbin Medical University (IRB3049723) and adhered to the “Guidelines for the Care and Use of Experimental Animals at Harbin Medical University.” All mice were housed in a temperature‐controlled room (23 °C ± 1 °C) with monitored humidity (65% ± 5%), under a 12‐h light/dark cycle.

Establishment of the HFD‐fed model: C57BL/6 mice and golden hamsters aged 6–8 weeks were fed an HFD for 12 weeks. The high‐fat feed, which was purchased from Diet Co., contained 60 kcal% fat, 20 kcal% carbohydrates, 1 kcal% phosphorus, and 20 kcal% protein.

*Ythdc1* knockdown experiment: *Ythdc1* flox^+/+^ mice were purchased from Cyagen Biosciences (Suzhou, China) and generated using the CRISPR/Cas9 system. Briefly, gRNA1 and gRNA2 were introduced into mouse zygotes to obtain homozygous hepatic knockout mice. These mice were crossbred with Cre‐expressing tool mice to produce homozygous liver‐specific *Ythdc1* knockout mice (*Ythdc1* cko). The *Ythdc1*‐loxp site was identified using the following primers:

Forward: 5′‐TCAAGGGGTGTATCATTTTATGGC‐3′

Reverse: 5′‐TGGCAGTCCATGTCTTTACTTCT‐3′.

*Ythdc1* overexpression experiment: 6‐week‐old C57BL/6 mice were injected with AAV8‐Ythdc1 or AAV8‐Control (HANBIO, Serotype: AAV8, Promoter: TBG, Titer: 1.8 × 10^12^ v. g mL^−1^, 100 µL per mouse, i.v.) through tail vein for 4 weeks. After injection, the mice were fed a normal diet (ND) or HFD for 12 weeks. Serum and liver tissues were harvested after 16 weeks for subsequent analysis.

*Ptpn22* knockdown experiment: *Ptpn22* conditional knockout (CKO) mice were generated by Cyagen Biosciences (Suzhou, China). Using the Cre‐loxP system, *Ptpn22* flox/flox mice were crossed with Alb‐Cre mice to produce liver‐specific *Ptpn22* knockout mice (*Ptpn22* flox/flox; Alb‐Cre, referred to as *Ptpn22* CKO). The *Ptpn22*‐loxp site was identified using the following primers:

Forward: 5′‐CTTCACTTCTCCAAAGTCACTGAAC‐3′

Reverse: 5′‐GCACATATGGAAGGACCTAAGAAG‐3′.

*Ptpn22* overexpression experiment: C57BL/6 mice aged 6–8 weeks were injected with AAV8‐*Ptpn22* or AAV‐control (HANBIO, Serotype: AAV8, Promoter: TBG, Titer: 1.8 × 10^12^ v. g mL^−1^, 100 µL per mouse, i.v.) through the tail vein for 4 weeks. After the injection, the mice were fed an ND or HFD for 12 weeks. At 16 weeks, body weight measurements and serum and liver tissues were collected for follow‐up studies.

MBZ treatment experiment: C57BL/6 mice fed an HFD for 4 weeks were divided into five groups:

1. ND + Saline group: Mice were fed an ND for 8 weeks and administered a daily gavage of 0.1 mL saline solution.
2. HFD + Saline group: Mice were fed an HFD for 8 weeks and gavaged daily with 0.1 mL saline solution.
3. HFD + MBZ (30 mg kg^−1^) group: Mice were fed an HFD for 12 weeks. Starting from the fourth week, the mice received a daily gavage of 30 mg kg^−1^ MBZ (HY‐17595, MedChemExpress, USA) for 2 months.
4. HFD + MBZ (60 mg kg^−1^) group: Mice were fed an HFD for 12 weeks. Starting from the fourth week, the mice received a daily gavage of MBZ (60 mg kg^−1^) for 2 months.
5. HFD + Atorvastatin (20 mg kg^−1^) group: Mice were fed a HFD for 8 weeks and received a daily gavage of atorvastatin (ATV; Pfizer, H2005140) at 20 mg kg^−1^ for 2 months.

### Human Liver Samples

The use of all human samples in this study was approved by the Ethics Committee of Harbin Medical University (Harbin, China; project license number: IRB‐AF/SC‐12/03.0). Liver tissue biopsies were obtained from patients diagnosed with fatty liver disease. Control liver samples were collected from healthy tissues adjacent to cancerous lesions. All participants provided written informed consent prior to sample collection.

### Cell Treatment

All cell lines were cultured at 37 °C in Dulbecco's modified Eagle's medium (DMEM; Gibco) supplemented with 10% fetal bovine serum (FBS; Gibco) and 1% penicillin‐streptomycin (Gibco). The cells were incubated in a 5% CO2 atmosphere for the subsequent experiments. HepG2, L02, and THLE‐2 cell lines were purchased from the following sources: HepG2 from Procell Life Science & Technology Co., Ltd.; L02 from the Type Culture Collection of the Chinese Academy of Sciences, Shanghai, China; and THLE‐2 from iCell Bioscience Inc.

### Cell Transfection and Treatment

HepG2, L02, and THLE‐2 cells were transfected with siRNA and plasmids using the jetPRIME transfection reagent (Polyplus) in accordance with the manufacturer's protocol.

The sequences for human si‐YTHDC1 were

Sense: 5′‐CGACCAGAAGAUUAUGAUATT‐3′.

Antisense: 5′‐UAUCAUAAUCUUCUGGUCGAC‐3′.

The sequences for human si‐PTPN22 were

Sense: 5′‐GAAUUGAUACAGCAGAGAGAAdTdT‐3′.

Antisense: 5′‐UUCUCUCUGCUGUAUCAAUUCdTdT‐3′.

The sequences for human si‐LDHA were

Sense: 5′‐GGCAAAGACTATAA‐3′.

Antisense: 5′‐TTATAGTCTTTGCC‐3′.

The sequences for human si‐AARS1‐2 were

Sense: 5′‐CAUCGGUCUAGAACCACUAdTdT‐3′.

Antisense: 5′‐UAGUGGUUCUAGACCGAUGdTdT‐3′.

Plasmids for human YTHDC1 (VB211229‐1194nye), PTPN22 (VB230503‐1450scj), NLRP3 (VB240820‐1701byr), AARS1 (VB250420‐1247urg), Flag‐YTHDC1 (VB250226‐1769zeq), Flag‐YTHDC1‐K58A (VB250226‐1777pnr), Flag‐YTHDC1‐K72A (VB250226‐1782wbu), Flag‐YTHDC1‐K82A (VB250226‐1784ntb), Flag‐YTHDC1‐K96A (VB250226‐1785tnm), Flag‐YTHDC1‐K127A (VB250226‐1788gfq), Flag‐YTHDC1‐K132A (VB250226‐1791jnk), Flag‐YTHDC1‐K565A (VB250226‐1796 mgx), and NLRP3‐Y861F (VB240820‐1731npn) were purchased from VectorBuilder (Guangzhou, China). When cells reached 80% confluence, OA (O1383‐1G, Sigma, USA) was added at a final concentration of 0.4 mmol L^−1^ to simulate a high‐fat in vitro model. The cells were treated for 24 h before further experimentation.

### L‐Lactylation Modification Omics

Proteins were extracted from the cells and digested with trypsin, and peptides were dissolved in IP buffer (100 mM NaCl, 1 mM ethylenediaminetetraacetic acid [EDTA], 50 mM Tris‐HCl, 0.5% NP‐40, pH 8.0). After overnight incubation with PTM1404 antibody‐coupled resin (PTM Bio) at 4 °C, the resin was washed (IP buffer ×4, H_2_O ×2), and the bound peptides were eluted with 0.1% TFA (3×), vacuum‐dried, and desalted using C18 ZipTips. Peptides were separated using a NanoElute UHPLC system (gradient: 6%–80% B; 450 nL min^−1^; mobile phase A: 0.1% FA/2% ACN; B: 0.1% FA/ACN) and analyzed on a timsTOF Pro mass spectrometer (1.6 kV ion source) in dia‐PASEF mode (MS1:100–1700 m/z; MS2:425–1025 m/z, 25 m/z windows).

### Histological Analysis

Liver samples were fixed in 4% paraformaldehyde for 24 h and embedded in paraffin for histological analysis. Tissue sections (4 µm thick) were prepared and stained with H&E. The staining procedure included staining with hematoxylin for 10 min, differentiation in differentiation solution for 3 min, staining with eosin dye for 30 s, and dehydration, clearing, and sealing of the slides.

For lipid accumulation analysis, frozen liver sections (thickness, 8 µm) prepared using optimal cutting temperature (OCT) embedding were stained with Oil Red O (Nanjing Jiancheng, D027‐1‐2). Histological features of the tissues were examined and imaged using an optical microscope (Leica, Germany). For Oil Red O staining in cells, cells were fixed with paraformaldehyde; the fixed cells were rinsed with 60% isopropyl alcohol; stained with Oil Red O solution for 5–10 min; and rinsed with distilled water and counterstained with hematoxylin. Differentiation was performed using 1% hydrochloric acid for rapid differentiation and staining.

### Co‐Immunoprecipitation Assay

To verify the interactions between proteins, the cells were lysed using lysis buffer. Protein complexes were captured using antibodies specific to PTPN22 (A1406, Abclonal, China), NLRP3 (A12694, Abclonal, China), and rabbit immunoglobulin G (IgG), which served as the negative control. Protein A/G agarose beads (MCE; HY‐K0202) were used to precipitate the antibody‐bound protein complexes. The precipitated complexes were subjected to further analysis to confirm the protein interactions.

### Immunoprecipitation Assay

Proteins were extracted from the cells using radioimmunoprecipitation assay (RIPA) buffer, and a portion of the lysate was retained as the input. The magnetic beads were incubated with primary antibodies overnight at 4 °C. The antibodies used for the immunoprecipitation assay were anti‐Ythdc1 (1:1000; 14392‐1‐AP; Proteintech, China), anti‐Ptpn22 (1:1000; A1406; Abclonal, China), and anti‐NLRP3 (1:1000; A12694; Abclonal, China). Protein A/G PLUS‐agarose was combined with the protein lysate and incubated overnight at 4 °C to remove proteins that specifically bound to the beads. The loading buffer was then added to the protein lysate, heated to 100 °C, and maintained for 10 min. The prepared samples were used for subsequent western blot analyses.

### Immunofluorescence Staining

At 48 h after inoculation in 24‐well plates, HepG2, Thle‐2, and L02 cells were washed three times with phosphate‐buffered saline (PBS). The cells in each well were fixed with paraformaldehyde for 15 min, followed by three additional washes with PBS. The cells were then permeabilized using a mixture of Triton (1139; BioFroxx, China), bovine serum albumin (BSA; BS114‐100 g; Biosharp, China), and PBS (G4207‐500ML, Servicebio, China) for 30 min at room temperature in the dark. After permeabilization, the cells were washed three times with PBS and blocked with goat serum (AR0009, Boster, China) for 1 h. The primary antibody against YTHDC1 (14392‐1‐AP, Proteintech, China) was applied, and the samples were incubated overnight at 4 °C. The following day, the secondary antibody was applied, and the samples were incubated for 1 h in the dark. The cells were then stained with 4′,6‐diamidino‐2‐phenylindole (DAPI; Beyotime Institute of Biotechnology, Shanghai, China) for 30 min and sealed. Stained samples were visualized using a laser scanning confocal microscope (Carl Zeiss) to quantify the stained areas.

### MeRIP and RNA Sequencing

For the meRIP experiment, the enriched poly(A)+ RNA was fragmented to ≈100 nucleotides by treatment with 20 mM ZnCl_2_ at 95 °C for 5–10 min. A portion of the fragmented RNA (10%) was retained as the “Input” control, while the remaining RNA was used for m^6^A immunoprecipitation. Poly(A)+ RNA was incubated with a specific anti‐N6‐methyladenosine (m6A) polyclonal antibody (Synaptic Systems, item #202 003) and RNasin (Promega, item #N2615) at a final concentration of 40 U µL^−1^ at 4 °C for 2 h. The m^6^A antibody specifically binds to m^6^A‐modified RNA. The RNA‐antibody complex was immunoprecipitated using Protein G magnetic beads (Thermo Fisher, item #88 848) by incubation at 4 °C for 1 h, ensuring efficient capture of the m6A‐modified RNA. The meRIP‐seq experiment, library preparation, high‐throughput sequencing, and data analysis were performed by SeqHealth Technology Co., Ltd. (Wuhan, China).

### Radioimmunoprecipitation Assay

The radioimmunoprecipitation (RIP) assay was performed according to the manufacturer's protocol using an RIP kit (3 996 278; Sigma‐Aldrich, USA). Briefly, the procedure was as follows:

Cell preparation: Liver tissue was homogenized in a cell suspension using a homogenizer. The cells were then lysed in lysis buffer for 5 min.

Antibody incubation: The lysate was incubated with 50 µL of magnetic beads pre‐conjugated with antibodies against YTHDC1 or IgG (used as a control) and incubated overnight at 4 °C.

Immunoprecipitation: The immune complex was briefly centrifuged and washed six times with RIP wash buffer to remove nonspecifically bound materials.

RNA purification and analysis: RNA was purified from the immunoprecipitated complex, and the final product was analyzed by quantitative RT‐PCR (qRT‐PCR) to assess the presence and levels of the target RNA.

### Actinomycin D and Cycloheximide Assay

HepG2 cells were treated with actinomycin D (HY‐17559; Med Chem Express, USA) or dimethyl sulfoxide (DMSO) as a control. Total RNA (1 µg) was incubated with 1 U RNase R at 37 °C for 10 min. After treatment with actinomycin D or RNase R, RNA stability was assessed using qRT‐PCR.

HepG2 cells were treated with the eukaryotic protein synthesis inhibitor cycloheximide (HY‐12320; Med Chem Express, USA) at a final concentration of 20 µg mL^−1^ in the culture medium. The cells were exposed to cycloheximide for 0, 6, or 12 h to investigate its effects on protein synthesis and RNA stability.

Serum and tissue levels of TG, TC, LDL‐C, ALT, and AST TC (Nanjing, Jiancheng, A111‐1‐1), TG (Nanjing, Jiancheng, A110‐1‐1), LDL‐C (Nanjing, Jiancheng, A113‐1‐1), ALT (Nanjing, Jiancheng, C009‐2‐1) and AST (Nanjing, Jiancheng, C010‐2‐1) kits were purchased from Nanjing Jiancheng Bioengineering Institute, Nanjing, China. The levels of these markers were measured in accordance with the manufacturers’ instructions.

### Western Blot Analysis

Liver tissues and cultured cells were lysed using RIPA buffer (Beyotime, China) supplemented with 1% protease inhibitors (Roche, Switzerland). The lysates were subjected to ultrasonication to extract the protein supernatants. The protein concentration was measured using an enzyme‐labeled instrument. Proteins were separated using sodium dodecyl sulfate‐polyacrylamide gel electrophoresis (SDS‐PAGE) and transferred onto polyvinylidene difluoride (PVDF) membranes. The membranes were blocked and incubated with primary antibodies overnight at 4 °C, followed by incubation with secondary anti‐rabbit antibodies (1:10 000) for 50 min at room temperature. Protein bands were analyzed using Image Studio Software.

The primary antibodies used included anti‐YTHDC1 (1:1000; 14392‐1‐AP; Proteintech, China), anti‐PTPN22 (1:500; A1406; Abclonal, China), anti‐Pan‐Phospho‐Tyrosine (1:1000; AP0905; Abclonal, China), anti‐NLRP3 (1:1000; A12694; Abclonal, China), and anti‐β‐Actin (1:10 000; AC026; Abclonal, China).

### RNA Isolation and qRT‐PCR

TRIzol reagent (Invitrogen, Carlsbad, CA, USA) was used to extract RNA from the tissues or cells. Complementary DNA (cDNA) was synthesized by reverse transcription using a kit from Toyobo (Japan). SYBR Green (Toyobo) was added to prepare a reaction mix with the primers, and the relative mRNA expression levels were calculated by detecting the cycle threshold (Ct) values using a quantitative PCR system. The mRNA levels of YTHDC1, PTPN22, LDHA, 18S rRNA, and glyceraldehyde‐3‐phosphate dehydrogenase (GAPDH) were analyzed using the following primers:

**Table:** 

Genus	Name	Primer	Sequence(5′‐3′)
Homo	YTHDC1	FP	ACTGTATGGCAGTGTTGTAG
		RP	CACGCTACTGAACATAGGAA
Homo	PTPN22	FP	TACAAAGAATCCACCTGACT
		RP	CAATTTGCCCTATTGGACTT
Homo	18S	FP	CCTGGATACCGCAGCTAGGA
		RP	GCGGCGCAATACGAATGCCCC
Homo	GAPDH	FP	AAGAAGGTGGTGAAGCAGGC
		RP	TCCACCACCCTGTTGCTGTA
Homo	LDHA	FP	GATTCAGCCCGATTCCGTTAC
		RP	GAGTCCAATAGCCCAGGATGTG
Mus	Ythdc1	FP	GTCCACATTGCCTGTAAATGAGA
		RP	GGAAGCACCCAGTGTATAGGA
Mus	Ptpn22	FP	CTTGGAATATGTAACGCACC
		RP	TTTGCTGGAAGTTTGTCAC

### Enzyme‐Linked Immunosorbent Assay

In accordance with the manufacturer's instructions, cells were collected to measure circulating levels of IL‐18 (MB‐2905A) and IL‐1β (MB‐2776A) using commercially available enzyme‐linked immunosorbent assay (ELISA) kits (Elabscience, Wuhan, China).

### Immunohistochemistry

Human tissue samples were embedded in paraffin, sectioned, and stained with the appropriate antibodies. Chemical reactions were used to enhance chromogenic agents, enabling the quantification of protein expression.

### Statistical Analysis

All data were expressed as mean ± standard error of the mean (SEM) from at least three independent experiments. Student's t‐test was used for comparisons between two groups. For comparisons involving multiple groups, one‐way analysis of variance (ANOVA) followed by Dunnett's post‐hoc correction was applied. Statistical analyses were conducted using GraphPad Prism 9.0 software (GraphPad Software, San Diego, California, USA). A *p* value < 0.05 was considered statistically significant.

## Conflict of Interest

The authors declare no conflict of interest.

## Author Contributions

F.Z., L.Z., and K.Z. contributed equally to this work. Y.B., and T.D. conceived the study and designed the experiments. Y.B., F.Z., L.Z., and K.Z. contributed to the methodology and performed data analysis; L.L. and Z.Y. collected clinical samples; K.L., F.Y., J.S., S.Z., H.F., Z.Y., C.W., H.D., and X.K. conducted cellular and molecular biological experiments; K.L., X.K., and K.Z. contributed to data curation and formal analysis; F.Z., L.Z., T.P., and H.D. performed the animal studies and analyzed the data; J.K. language polishing and grammatical corrections. All authors contributed to the original draft, editing, and revision. All authors reviewed and approved the manuscript.

## Supporting information



Supporting Information

## Data Availability

Research data are not shared.
