# Supplementary material for: Lactylation‐Driven YTHDC1 Alleviates MASLD by Suppressing PTPN22‐Mediated Dephosphorylation of NLRP3
Source: Adv Sci (Weinh). 2025 Nov 26;13(20):e10192. doi: 10.1002/advs.202510192 (PMC13067837; doi:10.1002/advs.202510192)
Supplement: Supplementary file 1 — Supporting Information [file ADVS-13-e10192-s001.docx]

**Supplementary Figure**


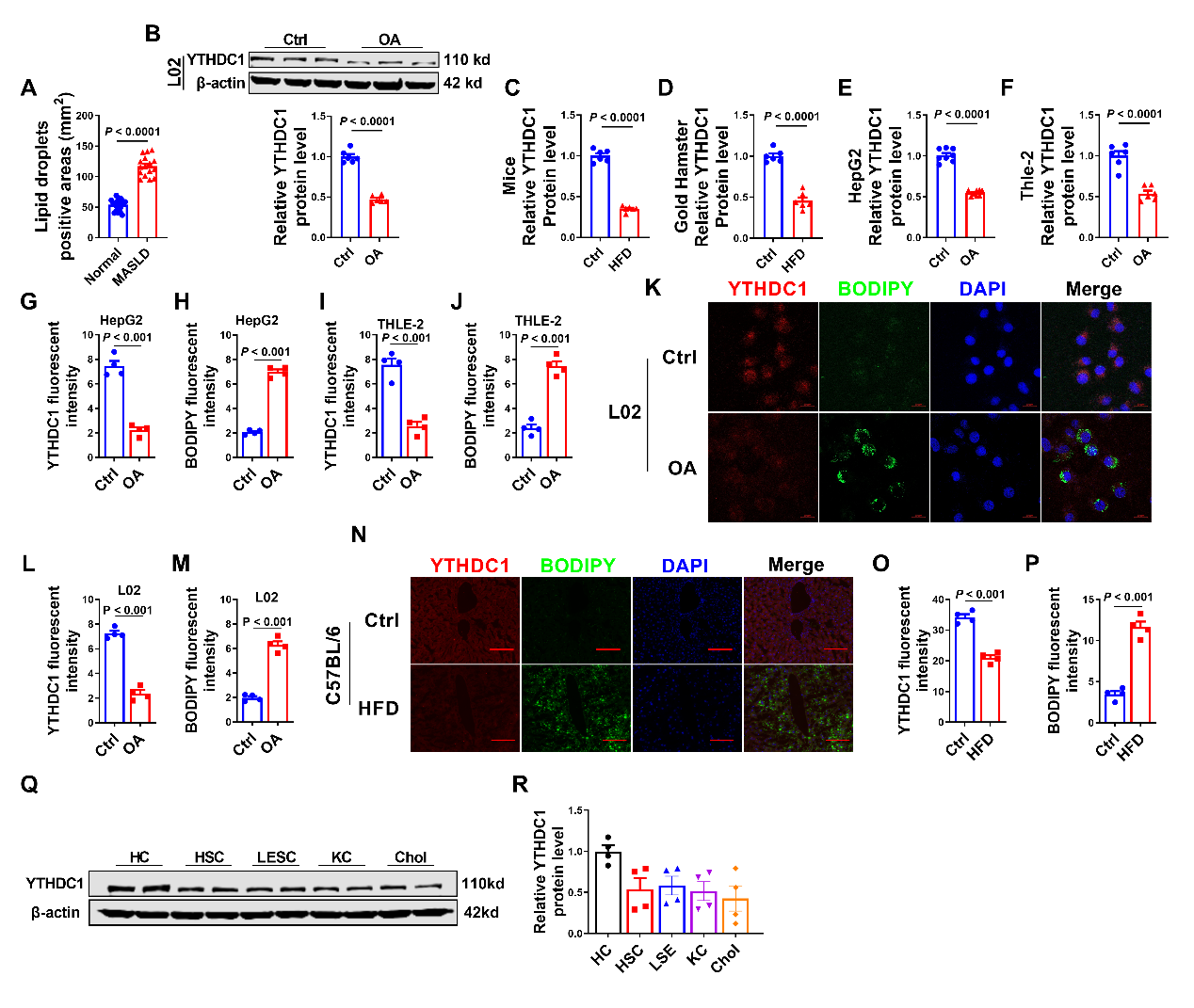


**Supplementary Figure 1** YTHDC1 is downregulated in MASLD. (A) Lipid droplets positive areas statistics datas of liver tissue and normal donor liver tissue in MASLD patients. n=16-21. (B) Western blot analysis of YTHDC1 protein level in L02 cells. n=6. (C-F) Western blot analysis of YTHDC1 protein levels in HFD-induced mice and golden hamster groups and at 24 h after OA induction in HepG2 and Thle-2 cells. n=6. (G-J) Immunofluorescence and BODIPY statistics datas of YTHDC1 expression in oleic acid-induced HepG2 and Thle-2. n=6. (K-P) Immunofluorescence and BODIPY representative images and statistics datas of YTHDC1 expression in HFD-induced mice and oleic acid-induced HepG2 and Thle-2. n=4. (Q-R) Protein Expression of YTHDC1 in liver various primary cells. n=4. Data are presented as mean±SEM.


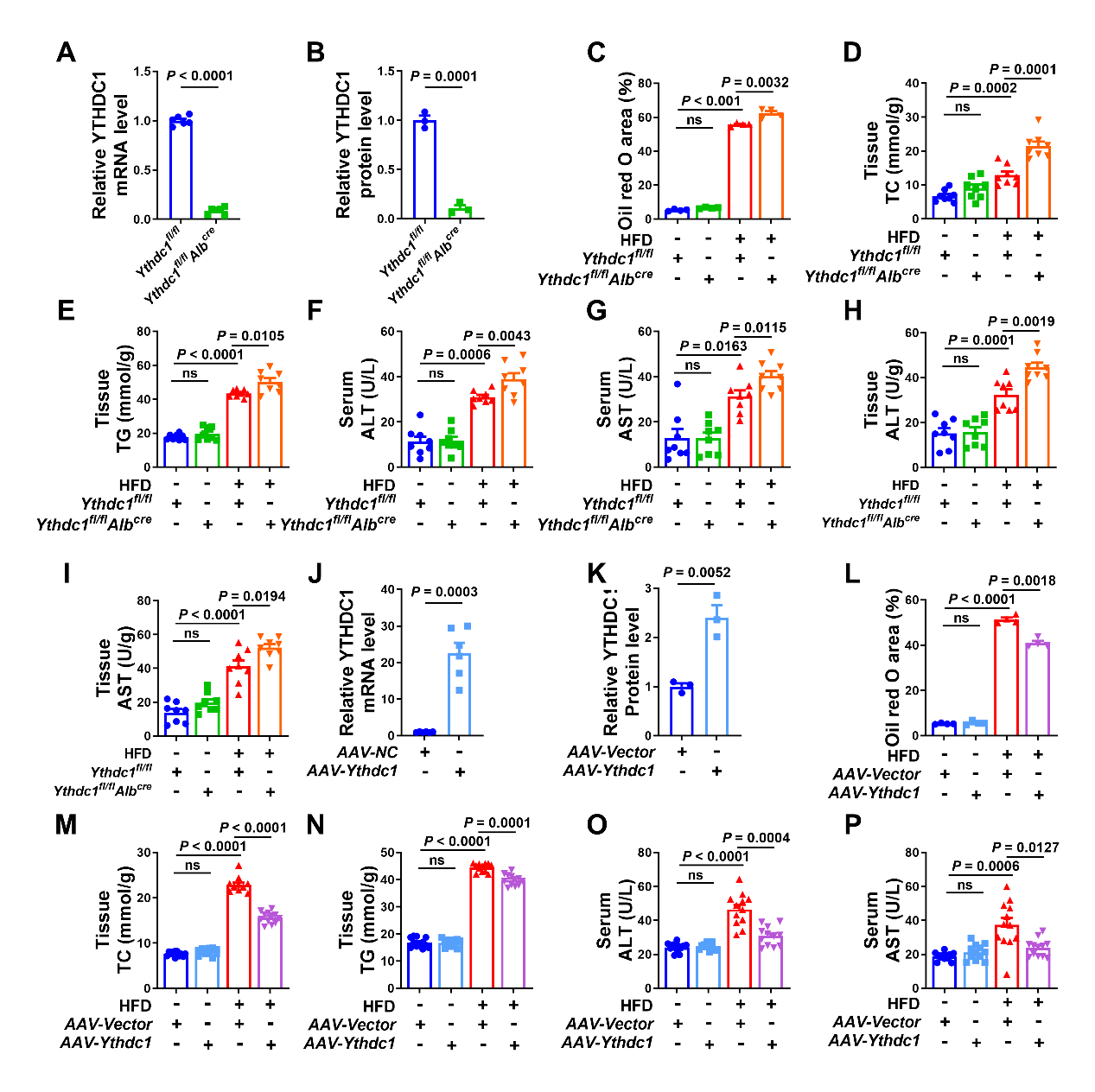


**Supplementary Figure 2** Modulating YTHDC1 expression regulates the progression of MASLD. (A, B) PCR and Western blot analysis of *Ythdc1* mRNA levels in the liver of *Ythdc1^fl/fl^* mice and *Ythdc1^fl/fl^Alb^cre^* mice. n=3-6. (C) Statistics datas of Oil Red O staining of liver of *Ythdc1^fl/f^* and *Ythdc1^fl/fl^Alb^cre^* mice after HFD feeding. n=4. (D-I) After 12 weeks of HFD induction, the levels of TC, TG, ALT and AST in tissues and the ALT and ALT level in serum of *Ythdc1^fl/fl^* and *Ythdc1^fl/fl^Alb^cre^* mice. n=8. (J, K) PCR and Western blot analysis of *Ythdc1* mRNA levels in the liver of *AAV-Ythdc1*mice and *AAV-Vector* mice. n=3-6. (L) Statistics datas of Oil Red O staining of liver of *AAV-Ythdc1*mice and *AAV-Vector* mice after HFD feeding. n=4. (M-P) After 12 weeks of HFD induction, the levels of TC and TG in tissues and the ALT and ALT level in serum of *AAV-Ythdc1*mice and *AAV-Vector* mice. n=12. Data are presented as mean±SEM.


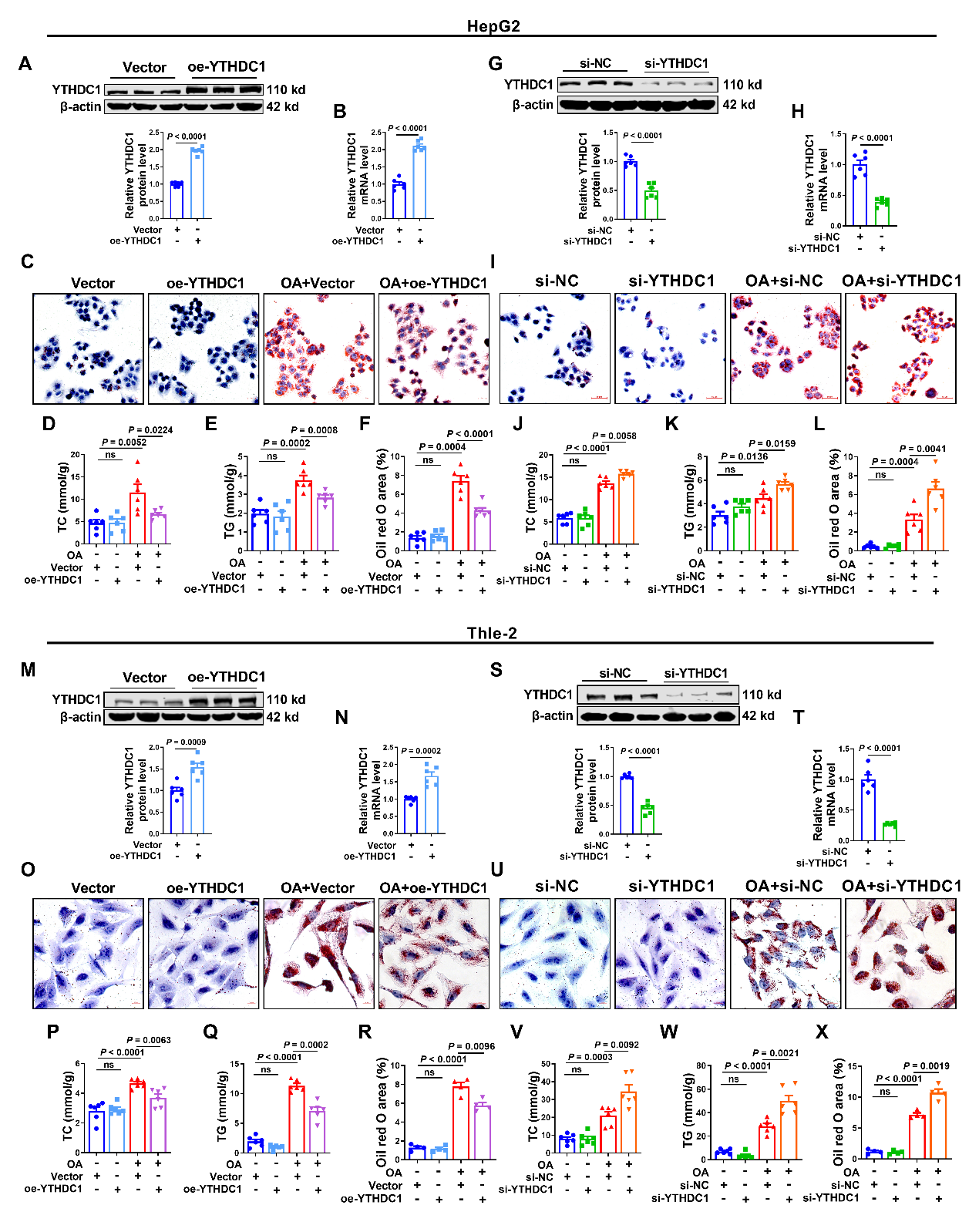


**Supplementary Figure 3** YTHDC1 regulates lipid accumulation in HepG2 and Thle-2 cells. (A) Western blot analysis of YTHDC1 protein levels in HepG2 cells transfected with oe-YTHDC1. n=6. (B) PCR analysis of *YTHDC1* mRNA level in HepG2 cells transfected with oe-YTHDC1. n=6. (C) and (F) Oil Red O staining representative images of oleic acid-induced HepG2 cells transfected with oe-YTHDC1. Scale bars=50µm. n=6. (D) and (E) Oleic acid-induced TC and TG levels in HepG2 cells transfected with oe-YTHDC1. n=6. (G) Western blot analysis of YTHDC1 protein level after transfection of HepG2 cells with si-YTHDC1. n=6. (H) PCR analysis of *YTHDC1* mRNA level in HepG2 cells transfected with si-YTHDC1. n=6. (I) and (L) Oil Red O staining representative images of oleic acid-induced HepG2 cells transfected with si-YTHDC1. Scale bars=50µm. n=6. (J, K) Oleic acid-induced TC and TG levels in HepG2 cells transfected with si-YTHDC1. n=6. (M) Western blot analysis of YTHDC1 protein levels in Thle-2 cells transfected with oe-YTHDC1. n=6. (N) PCR analysis of *YTHDC1* mRNA level in Thle-2 cells transfected with oe-YTHDC1. n=6. (O) and (R) Representative Oil Red O staining images of oleic acid-induced Thle-2 cells transfected with oe-YTHDC1. Scale bars=50µm. n=4. (P) and (Q) Oleic acid-induced TC and TG levels in Thle-2 cells transfected with oe-YTHDC1. n=6. (S) Western blot analysis of YTHDC1 protein levels after si-YTHDC1 transfection of Thle-2 cells. n=6. (T) PCR analysis of the *YTHDC1* mRNA level in Thle-2 cells transfected with si-YTHDC1. n=6. (U) and (X) Oil Red O staining representative images of oleic acid-induced Thle-2 cells transfected with si-YTHDC1. Scale bars=50µm. n=4. (V) and (W) Oleic acid-induced TC and TG levels in Thle-2 cells transfected with si-YTHDC1. n=6. Data are presented as mean±SEM.


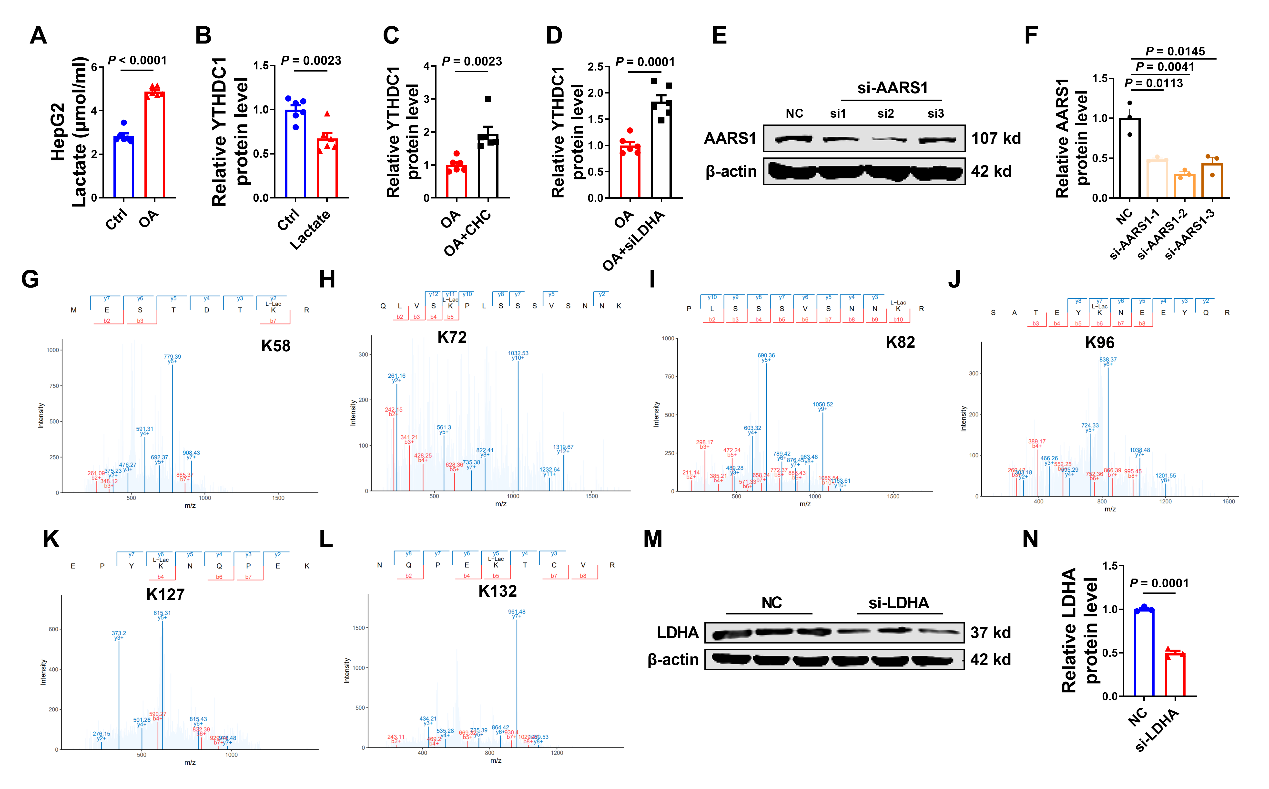


**Supplementary Figure 4** Multi-site lactylation suppresses YTHDC1 expression. (A) Statistical data of Lactate contents in HepG2 cells. n=6. (B-D) Statistical data of YTHDC1 protein levels in difference groups. n=6. (E) and (F) Western blot analysis of AARS1 in HepG2 cells. n=3. (G-L) lactylation sites of YTHDC1. (M) and (N) Western blot analysis of LDHA in HepG2 cells. n=3. Data are presented as mean±SEM.


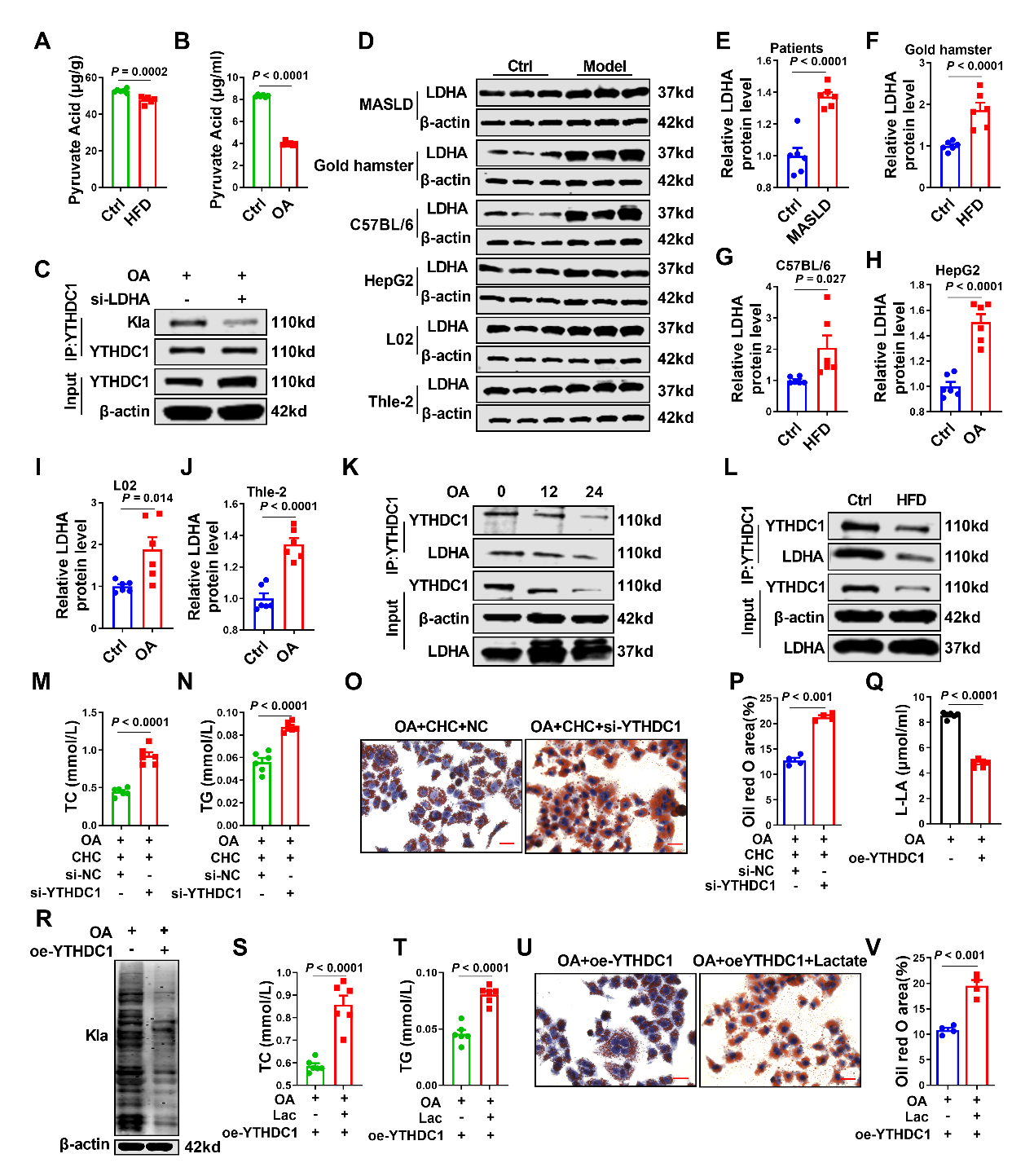


**Supplementary Figure 5** LDHA is a key mediator of YTHDC1 lactylation. (A, B) Statistical data of pyruvate contents in HepG2 cells. n=6. (C) HepG2 cell extracts were immunoprecipitated with an anti-YTHDC1 antibody and then immunoblotted with antibodies specific for Pan-Kla and YTHDC1. n=4. (D-J) Western blot analysis of LDHA protein levels in different groups. n=6. (K, L) HepG2 cell and mice liver extracts were immunoprecipitated with an anti-YTHDC1 antibody and then immunoblotted with antibodies specific for LDHA and YTHDC1. n=4. (M, N) Statistical data of TC and TG in HepG2 cells. n=6. (O) Oil Red O staining of HepG2 cell coverslips. n=4. (P) Statistical data on Oil red O. n=4. (Q) Statistical data of L-LA in HepG2 cells. n=6. (R) Western blot analysis of Pan-Kla in tissue and HepG2 cells. n=6. (S, T) Statistical data of TC and TG in HepG2 cells. n=6. (U) Oil Red O staining of HepG2 cell coverslips. n=4. (V) Statistical data on Oil red O. n=4. Data are presented as mean±SEM.

**
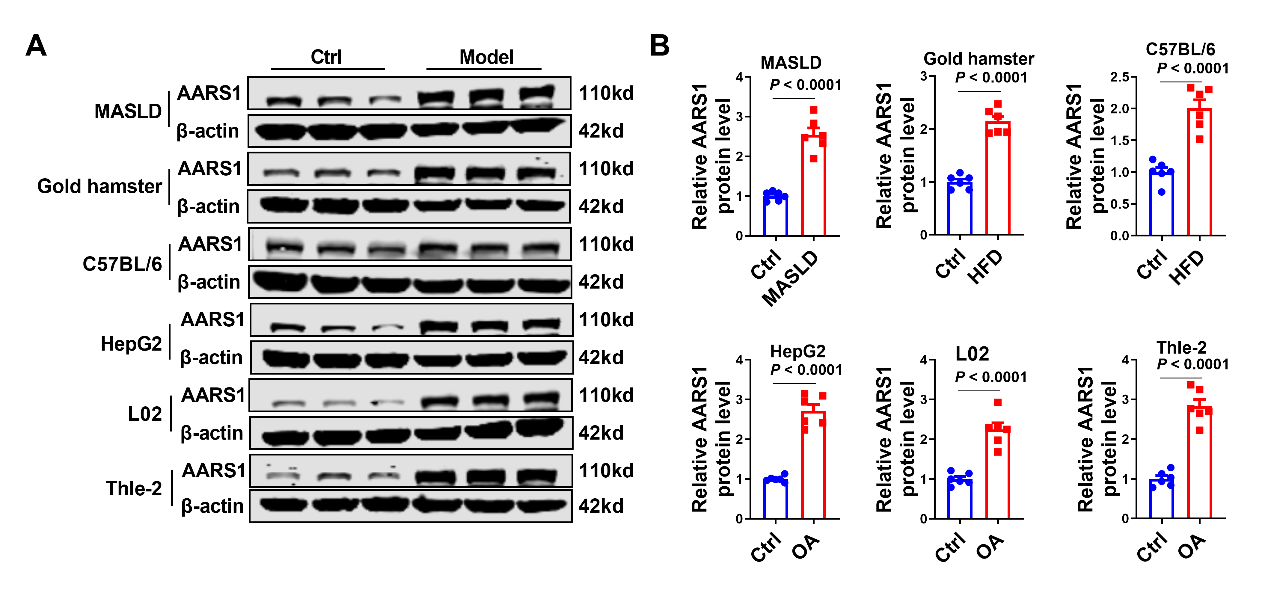
**

**Supplementary Figure 6** AARS1 protein expression of different models (A, B)Western blot analysis of AARS1 protein levels in different models. n=6. Data are presented as mean±SEM.


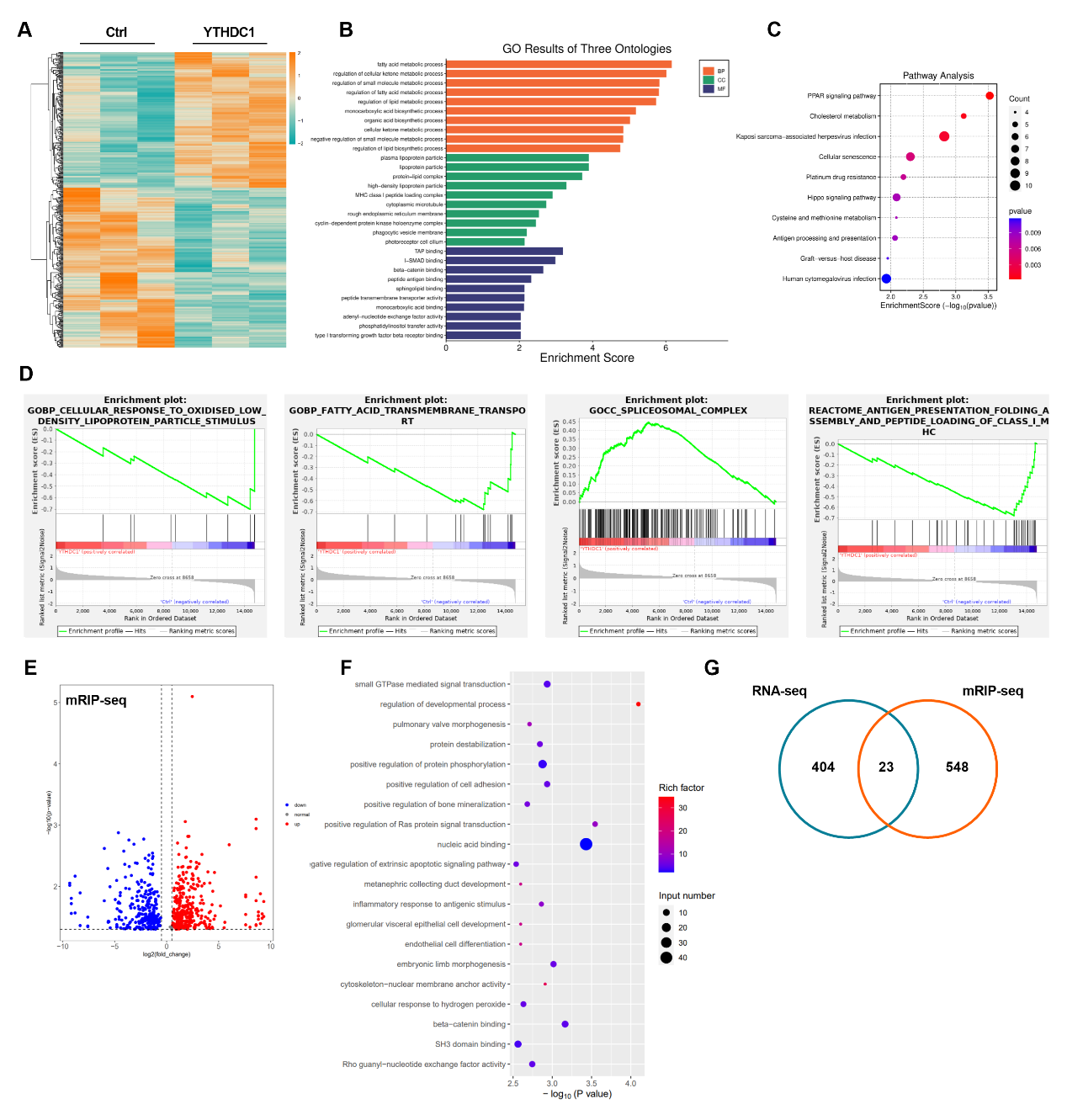


**Supplementary Figure 7** PTPN22 is a downstream target gene of YTHDC1. (A) Heatmap of differentially expressed genes after RNA-seq of tissues from the Ctrl and AAV-YTHDC1 groups of mice. (B) GO enrichment analysis of differentially expressed genes after RNA-seq of tissues from the Ctrl and AAV-YTHDC1 groups of mice. (C) KEGG pathway analysis of differentially expressed genes after RNA-seq of tissues from the Ctrl and AAV-YTHDC1 groups of mice. (D) GSEA analysis of differentially expressed genes after RNA-seq of tissues from the Ctrl and AAV-YTHDC1 groups of mice. (E) Volcano plot of differentially expressed genes after mRIP-seq of tissues from the Ctrl and AAV-YTHDC1 groups of mice. (F) KEGG pathway analysis of differentially expressed genes after mRIP-seq of tissues from the Ctrl and AAV-YTHDC1 groups of mice. (G) Venn diagram of differentially expressed genes analyzed by RNA-seq and mRIP-seq.


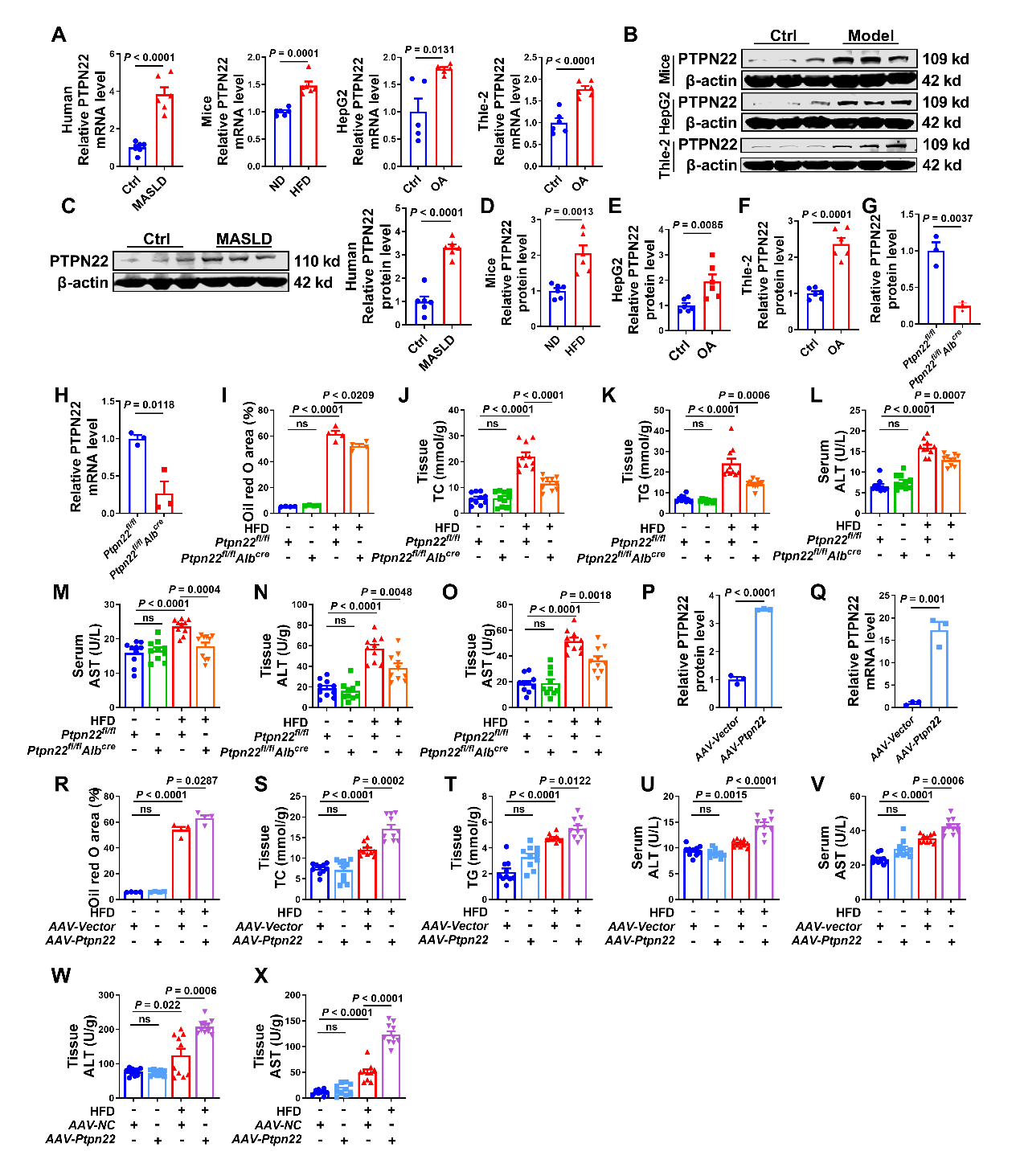


**Supplementary Figure 8** Upregulation of PTPN22 in MASLD. (A) PCR analysis of *PTPN22* mRNA levels in different models. n=5-6. (B-F) Western blot analysis of PTPN22 protein levels in different models. n=6. (G, H) Western blot and PCR analysis of *Ptpn22* mRNA level in *Ptpn22^fl/fl^* mice and *Ptpn22^fl/fl^Alb^cre^* mice. n=3. (I) Statistics datas of Oil Red O staining of liver of *Ptpn22^fl/fl^* mice and *Ptpn22^fl/fl^Alb^cre^* mice after HFD feeding. n=4. (J-O) After 12 weeks of HFD induction, the levels of TC, TG, ALT and AST in tissues and the ALT and ALT level in serum of *Ptpn22^fl/fl^* mice and *Ptpn22^fl/fl^Alb^cre^* mice. n=10. (P, Q) Western blot and PCR analysis of PTPN22 protein level in the liver of AAV-infected mice. n=3. (R) Representative images of liver sections of AAV-infected mice stained with HE and Oil Red O. n=4. (S-X) After 12 weeks of HFD induction, the levels of TC, TG, ALT and AST in tissues and the ALT and ALT level in serum of AAV-infected mice. n=10. Data are presented as mean±SEM.


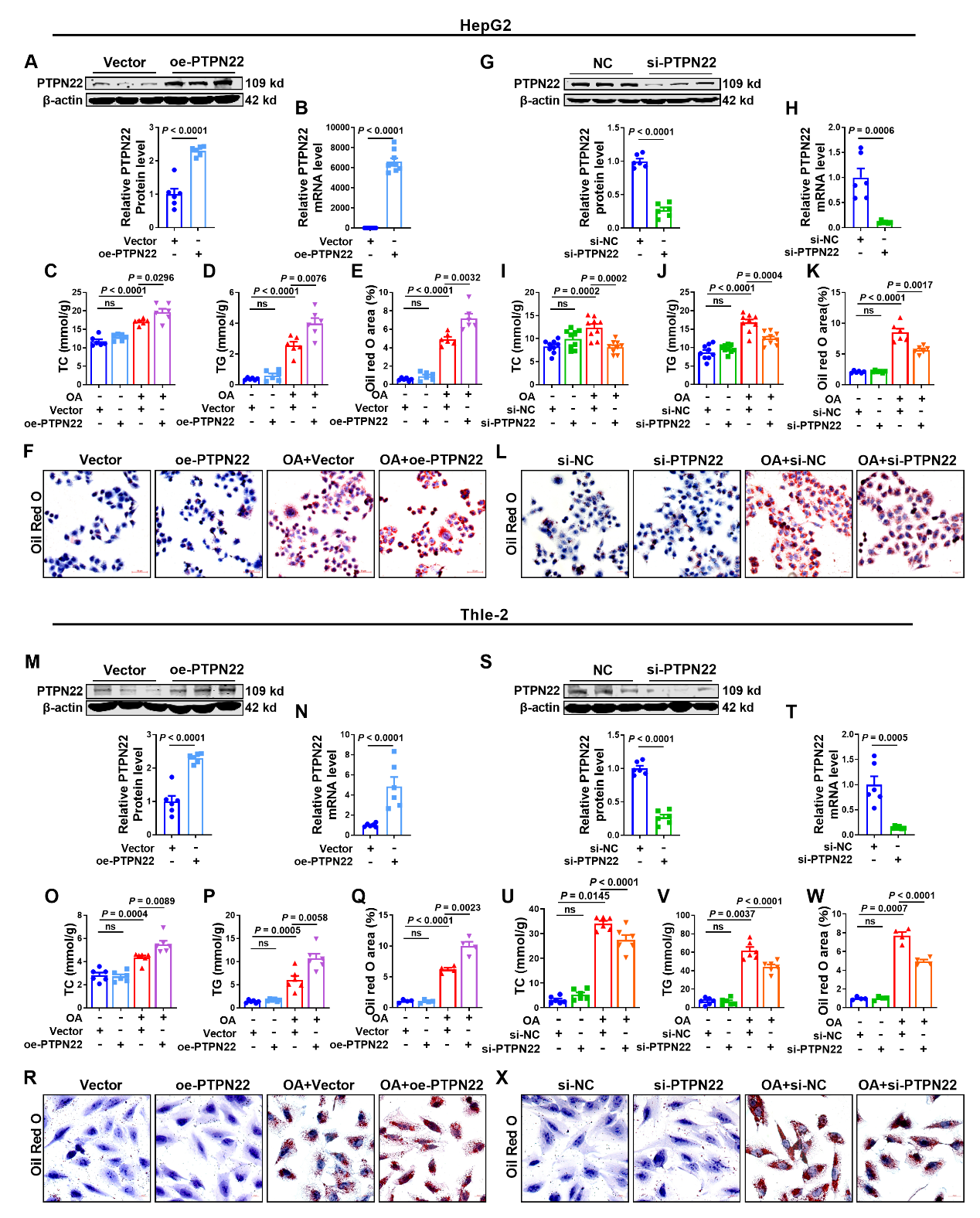


**Supplementary Figure 9** PTPN22 regulates lipid accumulation in HepG2 and Thle-2 cells. (A), (B), (G) and (H) Western blot and PCR analysis of *PTPN22* protein and mRNA levels in Vector, oe-PTPN22, si-NC and si-PTPN22 groups of HepG2 cells. n=6-9. (C), (D), (I) and (J) Statistical data of TC and TG in HepG2 cells. n=6-9. (E) and (K) Statistical data on Oil red O. n=6. (F) and (L) Oil Red O staining of HepG2 cell coverslips. n=6. (M), (N), (S) and (T) Western blot and PCR analysis of PTPN22 protein and mRNA levels in Vector, oe-PTPN22, si-NC and si-PTPN22 groups of Thle-2 cells. n=6. (O), (P), (U) and (V) Statistical data of TC and TG in HepG2 cells. n=6. (Q) and (W) Statistical data on Oil red O. n=4. (R) and (X) Oil Red O staining of HepG2 cell coverslips. n=4. Data are presented as mean±SEM.


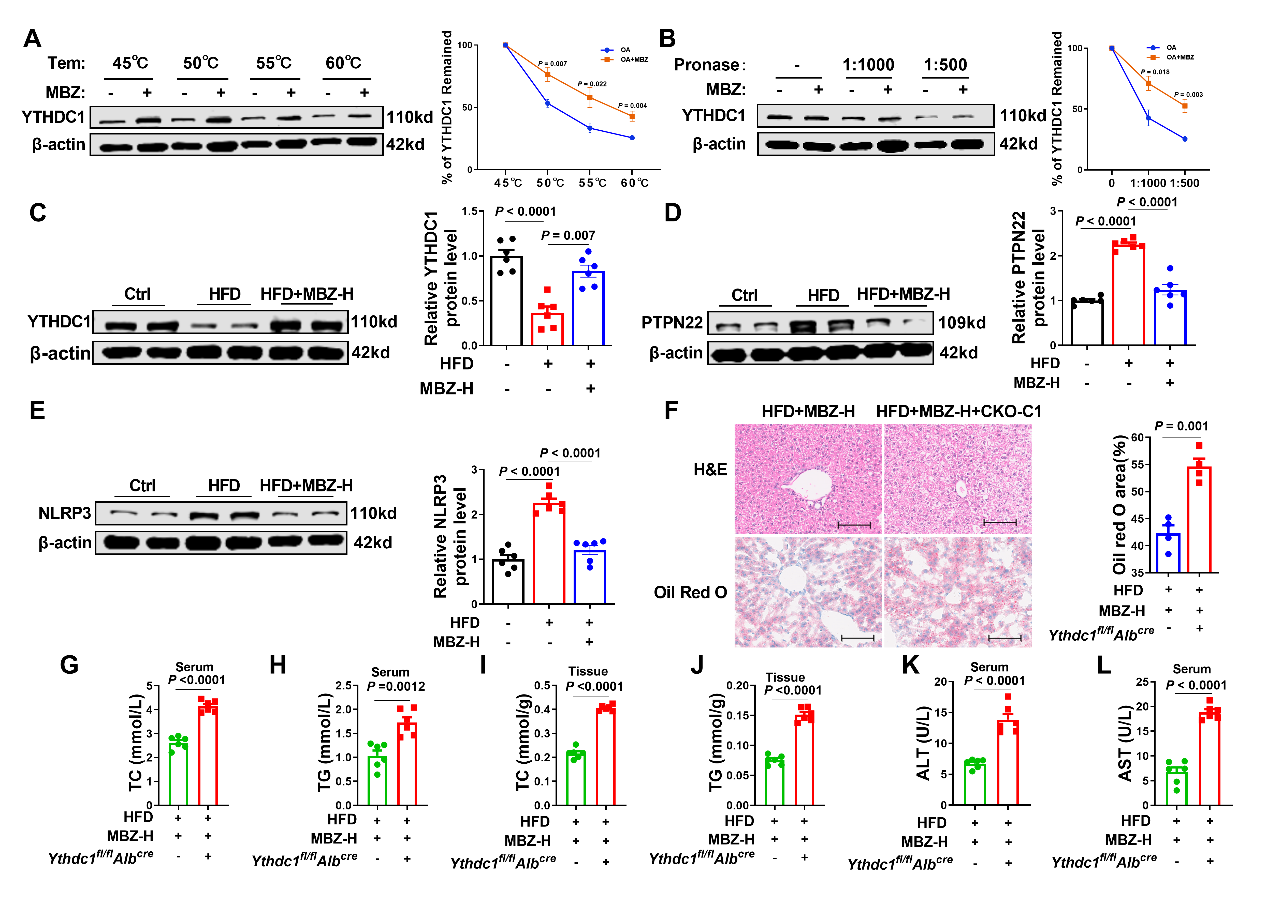


**Supplementary** **Figure 10** Mebendazole exerts its therapeutic effects against MASLD in a YTHDC1-dependent manner. (A, B) Western blot analysis revealing the increased thermal stability of MBZ binding to YTHDC1 in HepG2 cells. n = 3. (C-E) Western blot analysis of YTHDC1 protein levels in different models. n=6. (F) Statistics datas and representative images of HE staining and Oil Red O staining of liver sections. n=4. (G-L) Statistical data of TC, TG, ALT and AST contents in serum or tissue samples. n=6. Data are presented as mean±SEM.
